# Concordant Shared Transcriptomic Signatures and Candidate Regulatory Features in Chronic Lymphocytic Leukemia and Multiple Myeloma

**DOI:** 10.32604/or.2026.082424

**Published:** 2026-08-13

**Authors:** Abtin Tondar, David Hervás Marín, Laura Calvet Liñán, Asim Kumar Bepari

**Affiliations:** 1 Interuniversity Doctoral Program in Bioinformatics, Department of Computer Science, Multimedia and Telecommunication, Universitat Oberta de Catalunya (UOC), Barcelona, Spain; 2 Stanford Deep Data Research Computing Center, Stanford University, Stanford, CA, USA; 3 Department of Applied Statistics and Operations Research and Quality, Universitat Politècnica de València (UPV), Valencia, Spain; 4 Telecommunications and Systems Engineering Department, Universitat Autònoma de Barcelona (UAB), Sabadell, Spain; 5 Department of Pharmaceutical Sciences, North South University (NSU), Dhaka, Bangladesh

**Keywords:** Chronic lymphocytic leukemia, multiple myeloma, differential gene expression, transcriptomics, coexpression network, transcriptional regulation

## Abstract

**Background:** Chronic lymphocytic leukemia (CLL) and multiple myeloma (MM) are B-cell malignancies with distinct cellular origins and microenvironmental dependencies. We aimed to identify concordant transcriptomic signatures and candidate transcriptional regulatory features between CLL CD19-positive B cells and MM-associated bone marrow-derived mesenchymal stromal cells (MSCs). **Methods:** Public Gene Expression Omnibus bulk RNA sequencing datasets were analyzed separately within each context using DESeq2. Differentially expressed genes (DEGs) were defined using adjusted *p*-value < 0.05 and absolute log_2_ fold change > 1. Cross-disease analyses assessed overlap, directionality, log_2_ fold-change concordance, expressed-gene background-adjusted enrichment, coexpression structure, and transcription factor annotation. **Results:** We found a focused concordant gene-level signature shared across contexts. We identified 5965 DEGs in CLL and 1021 DEGs in MM; 323 were shared, and 262 were concordantly regulated, including 52 upregulated and 210 downregulated genes in both contexts. These genes showed strong log_2_ fold change concordance between CLL and MM. No Gene Ontology or Kyoto Encyclopedia of Genes and Genomes terms remained significant after expressed-gene background correction, supporting stronger gene-level than pathway-level evidence. Exploratory coexpression analysis identified PSMA3-AS1, SNORD58A, 100124516, MSS51, and 652966 as the top degree-ranked hubs and seven shared differentially expressed transcription factor candidates: MAFB, MYB, CCDC17, MYSM1, ZMAT1, ZNF491, and ZNF789. Expression-matched permutation analysis did not support global transcription factor enrichment. **Conclusion:** These findings support a cross-contextual concordant transcriptomic signature shared by CLL CD19-positive B cells and MM-associated MSCs, warranting validation in harmonized cohorts and experiments.

## 1 Introduction

Chronic Lymphocytic Leukemia (CLL) is the most prevalent leukemia in adults [1]. It is characterized by the clonal expansion of mature, functionally impaired B lymphocytes [2]. In this type of cancer, lymphocytes accumulate in tissues such as the peripheral blood, bone marrow, spleen [3], and lymph nodes [4]. The clinical manifestations of CLL can vary widely [5]. For instance, some patients experience an indolent disease that needs only minimal treatment, while others may develop aggressive forms of CLL with rapid progression [6].

Multiple microenvironmental and leukemia-intrinsic genetic factors contribute to the pathogenesis and clinical progression of CLL [7,8]. Recurrent cytogenetic lesions, including del(11q22 q23), del(13q14), and del(17p13), are established prognostic markers in CLL, with del(11q) and del(17p)/TP53 disruption generally associated with adverse outcomes, whereas isolated del(13q14) is typically associated with a more favorable prognosis [9,10]. Pathogenic alterations in TP53, ATM, and SF3B1 have also been linked to adverse clinical behavior, disease progression, treatment resistance, or shorter survival in CLL [11,12,13]. Moreover, B cell receptor (BCR) signaling and reciprocal interactions between CLL cells and the supportive tumor microenvironment promote leukemic cell survival, proliferation, disease progression, and therapeutic resistance [14,15].

In CLL, malignant B cells often express CD5, CD19, CD20, and CD23. Detecting this cell surface antigen panel can be used to diagnose CLL [16]. In addition, clinicians frequently use staging methods, such as the Rai and Binet systems, to classify CLL status based on parameters including lymphocytosis, lymphadenopathy, organomegaly, and cytopenias [16,17].

Multiple Myeloma (MM) is the second most prevalent hematologic cancer [18]. In MM, plasma cells in the bone marrow proliferate uncontrollably and produce excessive monoclonal immunoglobulins (M-proteins) [19]. Later, these proteins lead to organ dysfunction and failure [20]. MM is often diagnosed in older adults. The progression of MM in patients can vary vastly from very slow [21] to rapid, which exacerbates their medical complications [22].

Many factors can lead to MM. Previous studies have suggested that certain chromosomal translocations affecting the immunoglobulin heavy chain locus are associated with the progression of MM in some patients [23]. Moreover, chromosomal deletions, such as del(17p), which affect TP53, can exacerbate MM progression [24]. To date, several key regulatory genes that impact MM have been identified. For example, several mutations in BRAF [25], NRAS, and KRAS genes affect MM by increasing cell proliferation and survival [26]. The tumor microenvironment includes bone marrow stromal cells, cytokines, and extracellular matrix components. Many changes in this environment can also facilitate cell proliferation [27] and drug resistance [28] in MM patients. Furthermore, MM cells exploit signaling pathways, such as nuclear factor kappa B (NF-κB) [29] and interleukin 6 (IL-6)-mediated pathways [30], to promote survival and evade apoptosis.

The clinical symptoms of MM can include calcium elevation (hypercalcemia) [31], renal dysfunction [32], anemia [33], and bone lesions [34]. Bone marrow involvement in MM is a common feature due to increased osteoclast activation and reduced osteoblast function [35]. Diagnosis of MM is established through a combination of laboratory tests, imaging [36], and bone marrow biopsy [37]. For example, an elevated serum or urine M-protein [38], increased clonal plasma cells in the bone marrow [39], and evidence of end-organ damage [39] are important diagnostic markers in MM. The International Staging System (ISS) and Revised International Staging System ISS (R-ISS) are commonly used to classify MM severity based on factors such as β2-microglobulin, albumin levels, and genetic risk stratification [40].

Recent studies exploring new therapeutic strategies for leukemia and other hematologic malignancies emphasize the continuing need to identify molecular vulnerabilities that can support future treatment development [41,42]. Despite all the previous therapeutic advances, CLL and MM are still incurable [43]. In CLL, patients frequently relapse after treatment with B-cell receptor inhibitors or B-cell lymphoma 2 (BCL-2) antagonists such as Venetoclax, and resistance mechanisms commonly develop [44]. In the case of MM, patients typically experience multiple cycles of remission followed by relapse [45]. Despite the use of proteasome inhibitors, immunomodulatory agents, and monoclonal antibodies, many MM patients develop resistance to these therapeutic approaches [46]. Therefore, there is a pressing need to develop more effective treatment strategies applicable to both CLL and MM. Given the substantial research and development investment required to bring new therapeutic agents to market [47], understanding transcriptomic overlap between CLL and MM and identifying shared candidate regulatory features may help prioritize molecular vulnerabilities for future investigation [48].

Both CLL and MM are B-cell hematologic malignancies. However, they represent neoplastic transformations at fundamentally different stages of the B-cell developmental trajectory. These distinct biological frameworks profoundly shape their molecular profiles, progression, and response to therapies [49,50]. B-cell development is a tightly regulated process. It begins in the bone marrow and progresses through several stages, from immature B cells to naïve B cells. Subsequently, they differentiate into either memory B cells or antibody-producing plasma cells upon antigen exposure [51]. CLL arises from mature, antigen-experienced B cells. These cells typically resemble memory B cells in both phenotype and gene expression [52]. MM originates from terminally differentiated plasma cells that have undergone somatic hypermutation and class switching. At this stage, these cells are specialized for high-efficiency antibody production [53].

While both CLL and MM originate from the B-cell lineage, MM introduces an additional layer of complexity through its strong dependence on the bone marrow microenvironment [54], particularly its interactions with MSCs [55]. MM plasma cells reside primarily within the bone marrow niche and rely heavily on extrinsic signals from stromal cells [56], including MSCs, which secrete cytokines, chemokines [57], and growth factors [58] that promote tumor cell survival, proliferation [59], immune evasion [60], and resistance to therapy [61]. In contrast, although CLL cells can home to lymphoid tissues and bone marrow, they display a more autonomous survival program. They are less dependent on a single, structured microenvironment for progression [62]. Therefore, analyzing both cancers within a joint transcriptomic framework can help uncover how extrinsic versus intrinsic resistance mechanisms evolve across these two B-cell malignancies.

Analyzing transcriptome data from both CLL and MM within a single integrated study offers several methodological and discovery-related advantages, particularly in terms of data quality, analytical consistency, and the identification of shared molecular patterns [63]. Applying a unified computational workflow across both cancers reduces workflow-related variability in preprocessing, normalization, gene filtering, statistical modeling, and downstream interpretation [64]. This is important because differences in preprocessing, normalization strategy, and differential expression methodology across independent studies can introduce analytical inconsistencies and obscure biologically meaningful transcriptomic patterns [65]. In the present study, CLL and MM datasets were therefore processed using the same transcriptomic analysis framework to improve comparability and support a consistent summary-level interpretation of disease-associated signatures [66]. However, a unified workflow cannot eliminate dataset-specific, cohort-specific, or cell type-specific effects; accordingly, disease-versus-control comparisons were modeled within each dataset, and cross-disease findings were interpreted cautiously at the shared signature and pathway levels. This integrated approach can help identify convergent molecular signatures, co-expression patterns, and candidate regulatory programs that may be less apparent when CLL and MM are examined in isolation [67,68].

In this study, the unified analysis of CLL CD19-positive B cells and MM-associated MSCs provides a framework for identifying disease-associated transcriptomic signals that may be shared across distinct but related hematologic cancer contexts. Rather than directly comparing malignant cells from both diseases, our approach first defined disease versus matched-control expression changes within each dataset and then evaluated cross-disease overlap, directionality, log_2_ fold change concordance, coexpression structure, and candidate transcriptional regulatory features. This design allows the shared signals to be interpreted as cross-contextual transcriptomic patterns spanning CLL malignant B cell biology and the MM supportive stromal compartment, while avoiding claims of identical tumor-intrinsic mechanisms.

Although these findings do not establish validated therapeutic targets, they can support future target prioritization by identifying concordantly regulated genes, exploratory coexpression hubs, and shared differentially expressed TF candidates that may warrant further validation. Such candidates may help guide future studies focused on disease biology, microenvironmental dependency, and possible shared regulatory features between CLL and MM. Therefore, this study provides an exploratory transcriptomic and regulatory framework for comparing CLL and MM across distinct cellular compartments and for generating hypotheses that can be tested in larger, harmonized cohorts and experimental systems. Our study jointly analyzes CLL using CD19-positive B cells and MM using bone marrow-derived MSCs to investigate shared and distinct disease-associated transcriptomic signatures, coexpression patterns, and candidate regulatory features across two related B cell malignancy contexts.

## 2 Materials and Methods

This study was a retrospective computational analysis of publicly available RNA sequencing datasets. No new wet-lab experiments were performed by the authors, and no physical experimental reagents, consumables, or laboratory instruments were used directly in this study. Therefore, source reporting focused on public data sources, sequencing platforms reported in the original datasets, software packages, computational tools, annotation resources, and web-based databases used for analysis.

### 2.1 Datasets

Raw count RNA sequencing datasets were obtained from the Gene Expression Omnibus [69] (GEO; National Center for Biotechnology Information, National Library of Medicine, National Institutes of Health, Bethesda, MD, USA) database [70]. For CLL, we selected GSE70830, which includes peripheral blood CD19-positive B cells from 10 CLL patients and 5 control individuals [71]. Additional clinical information and cohort selection details for the CLL samples are summarized in Supplementary Table S1. For MM, we selected GSE196297, which includes bone marrow-derived mesenchymal stromal cells (MSCs) from six MM patients and five control donors [72]. Therefore, the MM dataset represents MM-associated stromal and microenvironmental transcriptional changes rather than malignant plasma cell intrinsic expression. Both datasets were generated using Illumina HiSeq sequencing 2000 (SY-401-1001) and 4000 (SY-401-4001) platforms for the GSE70830 and GSE196297, respectively (Illumina, Inc., San Diego, CA, USA) [73].

#### Dataset Selection Rationale and Sample Size Considerations

Datasets were selected using biological and technical criteria aligned with the objectives of this retrospective transcriptomic discovery study. Human primary sample datasets generated by RNA sequencing were prioritized over microarray-based datasets because RNA sequencing offers a broader dynamic range and is better suited for differential expression analysis, transcriptomic concordance assessment, and coexpression network analysis. Datasets were required to include both disease and control groups and to provide count-level expression data suitable for raw count-based differential expression analysis. Datasets involving drug treatment, therapeutic exposure, experimental stimulation, cell line manipulation, or other external perturbations were not selected because these factors could independently alter gene expression and confound disease-associated transcriptional signals.

We also prioritized datasets from biologically relevant cell sources that align with the analytical objective of this study. For CLL, GSE70830 was selected because it includes peripheral blood CD19-positive B cells from CLL patients and control individuals, enabling disease-versus-control comparison within the B cell compartment [74]. For MM, GSE196297 was selected because it contains bone marrow-derived MSCs from MM patients and control donors, enabling investigation of the MM supportive bone marrow microenvironment, which is critical for MM cell survival, proliferation, and therapy resistance [75]. Therefore, this MM dataset reflects MM-associated stromal and microenvironmental transcriptional alterations rather than malignant plasma cell intrinsic expression.

The cohort sizes in this study are modest. GSE70830 comprises 10 CLL samples and 5 control samples, while GSE196297 includes 6 MM samples and 5 control samples. Consequently, this analysis should be regarded as a retrospective discovery study based on public datasets rather than a prospectively powered clinical transcriptomic investigation. A formal prospective power analysis was not feasible because the study relied on pre-existing public datasets. Additionally, RNA sequencing power is influenced by gene-specific read abundance, dispersion, sequencing depth, effect size, multiple testing correction, and biological heterogeneity [76]. Biological replication is also a major determinant of power and reproducibility in RNA sequencing differential expression studies [77].

To reduce false-positive interpretations, a conservative differential expression threshold was applied: adjusted *p*-value < 0.05 and absolute log_2_ fold change > 1. Emphasis was placed on findings that were independently significant and, where applicable, directionally concordant across both disease contexts. Downstream analyses focused on DEG overlap, directionality classification, log_2_ fold-change concordance, pathway enrichment analysis using an explicitly defined expressed-gene background, FDR-corrected coexpression network analysis, hub gene prioritization, TF annotation, and permutation-based TF-overlap assessment. Although the modest sample size restricts broad generalizability, the selected datasets are suitable for hypothesis generation and for identifying candidate cross-contextual transcriptomic signals that require validation in larger, independent, and experimentally harmonized cohorts.

### 2.2 Pre-Processing of Raw RNA Counts

All post-iDEP computational analyses were conducted in R version 4.4.2 (R Foundation for Statistical Computing, Vienna, Austria) using RStudio Desktop version 2024.12.1+563 (Posit Software, PBC, Boston, MA, USA). Initial preprocessing, expression matrix processing, and exploratory visualization were performed using the Integrated Differential Expression and Pathway Analysis (iDEP 2.01; iDEP SDSU, South Dakota State University, Brookings, SD, USA) tool [78]. iDEP is an established RNA sequencing analysis platform used in published differential expression studies [79,80,81]. The iDEP software 2.01 was downloaded from its GitHub repository [82], installed, and run in RStudio version 2024.12.1+563 [83]. Raw count datasets were used as the input for preprocessing.

Genes were filtered to require a minimum expression level of 0.5 counts per million (CPM) in at least one library (N = 1). Although this N = 1 criterion is permissive, it was used only as an initial preprocessing filter to remove near-zero expressed features while retaining a broad transcriptomic background for exploratory and transformed expression-based analyses. To evaluate whether DEG-related results were sensitive to this filtering choice, we performed a stricter filtering sensitivity analysis in which the CPM cutoff was kept at 0.5, but the required number of libraries was increased from N = 1 to N = 3.

After filtering, transformed expression matrices were generated for exploratory and downstream analyses. Variance stabilizing transformation (VST) was used for mean variance assessment, scree plot generation, and PCA-based sample-level visualization. These VST-transformed matrices were used to evaluate whether the relationship between expression magnitude and variability was reduced and to assess the major sources of sample-level variation.

In parallel, processed expression matrices were generated using a log_2_(CPM + 4) transformation for expression-based downstream analyses. Any missing values in the processed expression matrices were imputed using the corresponding gene median expression value. These log_2_(CPM + 4)-processed matrices were used for the transformed expression-based downstream analyses, including sensitivity analyses and coexpression-based analyses. In contrast, primary differential expression testing was performed separately using raw count-based DESeq2 models, as described below.

In the present workflow, the VST-transformed matrices were used only for exploratory sample-level analyses, including mean-variance assessment, scree plot generation, and PCA-based visualization. In contrast, log_2_(CPM + 4)-transformed matrices were used for downstream expression-based analyses, including pseudocount sensitivity analysis, filtering sensitivity analysis, surrogate variable adjustment sensitivity analysis, and coexpression-based analyses. Primary DEG testing was performed separately using raw count-based DESeq2 models and did not use either the VST-transformed matrices or the log_2_(CPM + 4) processed matrices as the statistical input for differential expression testing.

PCA was performed [84] to visualize global sample-level variation. Specifically, the VST-transformed expression matrices were analyzed using the prcomp function in R, which projects samples onto orthogonal principal components that capture the major sources of variation in the dataset [85].

For processed expression-based downstream analyses, CPM values were transformed as log_2_(CPM + 4). The pseudocount of 4 was used to avoid undefined log values and to reduce instability among genes with very low CPM values. Although the default prior count commonly used in edgeR logCPM calculations is lower, a slightly larger pseudocount provides stronger moderation of low-abundance genes and reduces unstable log scale differences near zero expression. Therefore, this transformation is expected to have its greatest effect on lowly expressed genes, while having minimal influence on moderately or highly expressed genes. For example, relative to c = 2, using c = 4 changes the transformed value by 1.00 log_2_ unit at CPM = 0, approximately 0.22 log_2_ units at CPM = 10, and approximately 0.03 log_2_ units at CPM = 100. Importantly, this transformation was used for processed expression-based downstream analyses and was not used as the statistical basis for primary differential expression testing.

To clearly distinguish the analytical workflow, iDEP 2.01 was used only for initial raw count preprocessing, low expression filtering, transformation, processed matrix generation, and export of the VST and log_2_(CPM + 4) expression matrices. All post-iDEP analyses were conducted independently in R version 4.4.2 using RStudio Desktop version 2024.12.1+563. These R-based analyses included PCA using the prcomp function, mean variance assessment, scree plot generation, raw count-based DESeq2 differential expression testing, DEG overlap analysis, directionality classification, log_2_FC concordance analysis, GO and KEGG enrichment analysis, FDR-corrected coexpression network analysis, hub gene ranking, TF annotation, and permutation-based TF overlap assessment. Therefore, iDEP was not used for the primary DESeq2 differential expression modeling or for the downstream statistical analyses reported in the manuscript.

To assess the impact of the pseudocount choice on processed expression-based downstream analyses, a sensitivity comparison was performed using log_2_(CPM + 2). The effect of the pseudocount was most pronounced among genes with very low CPM values and diminished as expression abundance increased. Across the retained expression matrices, the median differences between pseudocount 4 and pseudocount 2 were minimal, ranging from 0.0020 to 0.0096 log_2_ units, and matrix-level correlations remained very high, ranging from 0.9989 to 0.9998. The stricter N = 3 filtering threshold further reduced pseudocount sensitivity by removing additional low-abundance genes. As expected, the pseudocount did not affect the primary raw count-based DESeq2 model. In the sensitivity comparison of downstream DEG-related outputs, the results were identical in the CLL N = 1, MM N = 1, and MM N = 3 settings, and highly concordant in the CLL N = 3 setting, among retained genes. These findings indicate that the pseudocount choice primarily affected low-abundance transformed expression values and did not materially alter the downstream biological interpretation.

### 2.3 DEG Analysis

#### 2.3.1 Within Dataset DESeq2 Differential Expression Analysis

For DEG analysis, raw count matrices from each GEO dataset were analyzed separately using the Bioconductor package DESeq2 version 1.44.0 in R version 4.4.2 (Bioconductor Project; catalog number not applicable; Buffalo, NY, USA), with gene identifiers standardized to Ensembl gene IDs where required [86]. Within each dataset, samples were assigned to disease or matched control groups based on their study-specific condition labels. A condition factor was then defined separately within each dataset in the sample annotation table. The design formula modeled raw counts as a function of condition within each dataset, rather than as a pooled cross-dataset model. Library size normalization and dispersion estimation were performed automatically by DESeq function. Disease-versus-control contrasts were extracted separately for each dataset using the DESeq2 contrast specification. Specifically, the CLL analysis compared CLL samples with control CD19-positive B cell samples within GSE70830, while the MM analysis compared MM-associated MSC samples with control MSC samples within GSE196297. DESeq2 default independent filtering was retained during result extraction to improve detection power by filtering genes with low mean normalized counts that provided limited evidence for differential expression. Cook’s distance-based outlier handling was also retained under DESeq2 default settings. Genes flagged by DESeq2 as affected by extreme count outliers or by independent filtering were assigned unavailable or adjusted *p*-values, where applicable, and were not considered significant. Adjusted *p*-values were calculated from Wald test *p*-values using the Benjamini-Hochberg false discovery rate correction. Genes were considered significantly differentially expressed if they had an adjusted *p*-value < 0.05, corresponding to false discovery rate control, and an absolute log_2_ fold change > 1, corresponding to at least a two-fold change on the linear scale.

#### 2.3.2 Low Expression Filtering Sensitivity Analysis

To evaluate whether DEG results were sensitive to the initial low-expression filtering threshold, we performed a filtering sensitivity analysis. The primary analysis retained genes with at least 0.5 CPM in at least one library. In the sensitivity analysis, the CPM cutoff was held fixed at 0.5, while the required number of libraries was increased from N = 1 to N = 3 under the same processed-expression setting with pseudocount = 4.

The potential interaction between the filtering strategy and the pseudocount value was considered because log_2_(CPM + 4) has its greatest influence on low-abundance genes. Increasing the filtering requirement from N = 1 to N = 3 removes genes detected in only one or two libraries, thereby reducing the contribution of the lowest-abundance features, where the pseudocount has the strongest effect on transformed expression values. Thus, the N = 3 sensitivity analysis was used to evaluate whether downstream DEG-related interpretations were robust to a stricter low-expression filter under the same transformed expression setting. Importantly, the log_2_(CPM + 4) transformation was not used as the statistical input for the primary DEG model, which was performed using raw count-based DESeq2 analysis.

Under the stricter filtering condition, raw count matrices were re-filtered separately for each dataset using the N = 3 criterion. DESeq2 analysis was then rerun independently for each dataset, including recalculation of library size normalization, size factors, dispersion estimates, and disease-versus-control contrasts under the stricter retained gene set. The same DEG thresholds were then applied: adjusted *p*-value < 0.05 and absolute log_2_ fold change > 1.

The N = 3 DEG lists were compared with the primary N = 1 DEG lists using explicit quantitative criteria. DEG overlap was defined as the number of genes significant under both filtering settings. DEG retention was calculated as the percentage of N = 1 DEGs retained under N = 3 filtering. Jaccard similarity was calculated as the size of the intersection divided by the size of the union of the N = 1 and N = 3 DEG sets. Direction concordance was defined as the percentage of overlapping DEGs with the same sign of log_2_ fold change under both filtering settings. Preservation of disease-associated expression patterns was evaluated quantitatively using DEG counts, overlap, DEG retention, Jaccard similarity, direction concordance, and Pearson and Spearman correlations of log_2_ fold change values among genes retained in both analyses.

#### 2.3.3 Cross-Cell Type Clarification

No direct DEG contrast was performed between CLL CD19-positive B cells and MM-associated MSCs. Differential expression was first estimated independently for each disease context by comparing each disease group with its own cell type-matched control group. Thus, the CLL DEG list was derived from CLL versus control CD19-positive B cells, whereas the MM DEG list was derived from MM-associated MSCs versus control MSCs. The cross-disease comparison was performed only after these within-context DEG analyses had been completed.

Shared significant DEGs were first defined as genes that satisfied the DEG criteria in both disease versus control comparisons, regardless of whether their direction of regulation was the same or opposite between CLL and MM. These shared significant DEGs were then classified according to directionality. Genes with positive log_2_ fold change values in both disease comparisons were classified as concordantly upregulated, genes with negative log_2_ fold change values in both comparisons were classified as concordantly downregulated, and genes with opposite log_2_ fold change signs between CLL and MM were classified as opposite direction shared DEGs. Opposite-direction shared DEGs were retained as a separate category because they identify genes that were significantly altered in both disease contexts but showed divergent regulatory direction. Analytically, this category was used to prevent total DEG overlap from being overinterpreted as concordant shared biology. Therefore, opposite-direction shared DEGs were reported as part of the total shared significant DEG pool but were not included in the concordantly regulated DEG set used for log_2_ fold-change concordance analysis, direction-specific enrichment testing, and coexpression network construction. Biologically, opposite-direction shared DEGs may reflect disease-specific regulation, differences between CD19-positive B cells and bone marrow-derived MSCs, distinct tissue-niche effects, compensatory transcriptional responses, or context-dependent regulation of the same genes. Accordingly, these genes were interpreted as discordant cross-context signals rather than evidence of a common transcriptional program between CLL and MM.

This design avoids a direct baseline comparison between CD19-positive B cells and MSCs during DEG identification. However, it does not eliminate cross-cell type interpretive limitations. Differences in cell lineage, tissue niche, differentiation state, baseline transcriptional activity, and microenvironmental activation may influence which genes are detected as differentially expressed across datasets. Therefore, the shared DEGs identified in this study were interpreted as cross-contextual disease-associated transcriptional signals rather than definitive evidence of identical tumor-intrinsic mechanisms in CLL and MM.

#### 2.3.4 Within Dataset DEG Clarification

No surrogate variable analysis (SVA) version 3.52.0 (Bioconductor Project; catalog number not applicable; Buffalo, NY, USA) or surrogate variable-based correction was applied before or during the primary raw count-based DESeq2 differential expression analysis. The primary DEG analyses were performed as within-dataset disease-versus-matched-control comparisons using raw count matrices. No explicit batch covariates were included in the primary DESeq2 models because suitable within-dataset batch metadata were not available. However, because SVA is designed to estimate latent sources of variation even when measured batch variables are unavailable, we evaluated the potential impact of latent variation through an exploratory SVA-based sensitivity analysis.

This sensitivity analysis was conducted separately within each GEO dataset and did not replace the primary raw count-based DESeq2 analysis. Processed expression matrices were analyzed using limma version 3.60.6 (Bioconductor Project; catalog number not applicable; Buffalo, NY, USA) models with and without surrogate variables. For each dataset, the unadjusted model was specified as expression ~ condition, and adjusted models were specified as expression ~ condition + surrogate variables.

To better reflect the data structure, we performed a data-driven estimate of the number of surrogate variables using the num.sv function from the sva package, with the primary N = 1 and pseudocount = 4 settings. Using the leek method, num.sv estimated two surrogate variables for the CLL dataset and two surrogate variables for the MM dataset. Using the Buja and Eyuboglu (BE) method as an additional reference, num.sv estimated four surrogate variables for the CLL dataset and two surrogate variables for the MM dataset. Because the cohorts were modest in size and excessive adjustment for surrogate variables can reduce degrees of freedom or remove disease-associated signal, the SVA analysis was retained as an exploratory sensitivity analysis rather than the primary DEG workflow. One- and two-surrogate-variable models were evaluated as conservative sensitivity settings, with emphasis on the two-surrogate-variable setting supported by the num.sv leek estimate in both datasets.

Robustness was assessed using DEG overlap, Jaccard similarity, Pearson and Spearman correlations of logFC values, t-statistic correlations, direction concordance, and top-ranked gene overlap. The analysis was repeated across N = 1 and N = 3 filtering and pseudocount 2 and 4 processed expression settings, with emphasis on the N = 1, pseudocount 4 setting used in the primary study workflow.

### 2.4 Identification of Shared Significant DEGs

We began with two datasets-specific DEG result tables, one from the CLL versus control CD19-positive B cell comparison and the other from the MM-associated MSC versus control MSC comparison. Each table contained gene identifiers, gene symbols, log_2_ fold changes, and adjusted *p*-values obtained through DESeq2. After loading these tables into R, column names were standardized, and the log_2_ fold change and adjusted *p*-value columns were verified as numeric. Rows with missing or nonconvertible values in the required DEG columns were removed to maintain data integrity.

The predefined DEG criteria were then applied separately to each dataset: adjusted *p* value < 0.05 and absolute log_2_ fold change > 1. Significant DEGs from the CLL and MM comparisons were intersected using standardized Ensembl gene IDs, while gene symbols were retained for annotation, visualization, and downstream interpretation. Before the intersection, Ensembl gene IDs from both DEG result tables were inspected for version suffixes. No versioned Ensembl identifiers were detected in the CLL or MM DEG result tables; therefore, the identifiers used for overlap analysis were already version independent. The overlap obtained using standardized Ensembl gene IDs was also consistent with the overlap obtained using trimmed gene symbols, and no symbol mismatches were detected among matched Ensembl IDs. No raw read-level reference remapping was performed at this stage because the analysis used DEG result tables derived from the public count matrices; this step was an identifier harmonization and verification procedure intended to avoid the loss of shared genes due to gene ID formatting or version discrepancies.

Genes that met the DEG criteria in both disease contexts were defined as shared significant DEGs, regardless of whether the direction of regulation was the same or opposite between diseases. This inclusive definition was used to document the full set of genes significantly altered in both comparisons before direction-based interpretation. Significantly shared DEGs were then separated into concordantly regulated and opposite direction categories. Opposite-direction shared DEGs were retained as a separate category because they were significant in both disease contexts but showed divergent regulatory directions. Analytically, this category was used to distinguish total DEG overlap from concordant shared regulation and to prevent the total shared DEG pool from being overinterpreted as evidence of common transcriptional mechanisms. These opposite-direction genes were not included in the concordantly regulated DEG subset used for log_2_ fold change concordance analysis, direction-specific enrichment testing, and coexpression network construction.

After the shared significant DEG set was identified, directionality was assigned based on the sign of the log_2_ fold change for each disease comparison. Genes with positive log_2_ fold change values in both CLL and MM were classified as concordantly upregulated shared DEGs. Genes with negative log_2_ fold change values in both comparisons were classified as concordantly downregulated shared DEGs. Genes with opposite log_2_ fold change signs between CLL and MM were classified as opposite-direction shared DEGs. This classification allowed the total shared DEG overlap to be distinguished from the subset of concordantly regulated shared DEGs used in subsequent concordance, coexpression, and regulatory analyses.

For visualization, the number of CLL-specific DEGs, MM-specific DEGs, total shared significant DEGs, concordantly regulated shared DEGs, and opposite direction shared DEGs was summarized in a Venn-style overlap plot generated in R using ggplot2 version 3.5.1 [87] (Comprehensive R Archive Network/R Foundation for Statistical Computing; catalog number not applicable; Vienna, Austria). All output tables, including the full shared significant DEG table, direction-classified shared DEG tables, and summary visualization files, were saved for downstream interpretation and reproducibility.

### 2.5 Shared DEG Log_2_FC Concordance Analysis

To further assess whether shared DEGs showed concordance beyond overlap alone, a log_2_ fold-change concordance analysis was conducted on the concordantly-regulated subset of shared DEGs. For each concordantly regulated shared DEG, the disease versus control log_2_ fold change from the CLL comparison was paired with the corresponding disease versus control log_2_ fold change from the MM comparison. Shared DEGs with opposite directions of regulation between CLL and MM were excluded from the same direction concordance analysis because they represented discordant cross-disease effects by definition.

Pearson correlation was used to evaluate linear concordance in log_2_ fold change magnitude, while Spearman rank correlation was used to assess rank-based concordance. Directional concordance was assessed by determining whether each shared DEG showed the same sign of log_2_ fold change in both disease contexts. Scatter plots were generated to visualize the relationship between CLL and MM log_2_ fold change values across the concordantly regulated shared DEG set. This analysis was used to quantify concordance in effect-size structure within the shared DEG subset, rather than to establish causal or mechanistic equivalence between CLL and MM.

### 2.6 Pathway Enrichment Analysis

Pathway enrichment analysis was performed to evaluate whether concordantly regulated shared DEGs between CLL and MM were overrepresented in Gene Ontology (GO; Gene Ontology Consortium; catalog number not applicable) categories [88] and Kyoto Encyclopedia of Genes and Genomes (KEGG; Kanehisa Laboratories; catalog number not applicable; Kyoto, Japan) pathways [89] when tested against an explicitly defined expressed gene background universe. The analysis was conducted in R using the clusterProfiler package version 4.12.6 (Bioconductor Project; catalog number not applicable; Buffalo, NY, USA) [90], with human gene annotation provided by org.Hs.eg.db version 3.19.1 (Bioconductor Project; catalog number not applicable; Buffalo, NY, USA) [91].

For enrichment analysis, the shared DEG set was restricted to genes that showed concordant direction of regulation in both disease contexts. Concordantly upregulated shared DEGs were defined as genes with log_2_ fold change > 1 and adjusted *p* value < 0.05 in both CLL and MM. Concordantly downregulated shared DEGs were defined as genes with log_2_ fold change < −1 and adjusted *p* value < 0.05 in both CLL and MM. Shared DEGs with opposite directions of regulation between CLL and MM were not included in the upregulated or downregulated enrichment tests.

In addition to the direction-specific enrichment analyses, we performed an additional enrichment analysis using the combined pool of concordantly regulated shared DEGs, including both concordantly upregulated and downregulated DEGs. This analysis was performed to evaluate whether pathway-level signals could be detected when concordantly altered genes with potentially different regulatory roles within pathways were analyzed together. The same explicit expressed gene background universe, Entrez ID mapping procedure, clusterProfiler settings, and Benjamini Hochberg multiple testing correction were used for the combined concordant DEG enrichment analysis.

To address the influence of the background gene universe on enrichment statistics, GO and KEGG enrichment analyses were performed using an explicitly defined expressed gene background rather than the default annotation database background. The background universe was constructed from post-filtered genes retained in both processed expression matrices used in the primary analytical setting, corresponding to N = 1 filtering and pseudocount = 4. Specifically, the retained gene identifiers from the GSE70830 and GSE196297 processed expression matrices were intersected to define the set of genes retained after filtering in both datasets and eligible for enrichment background inclusion. The GSE70830 processed matrix contained 20,117 post-filtered genes, and the GSE196297 processed matrix contained 18,700 post-filtered genes. Their intersection contained 16,316 common post-filtered input identifiers.

These input identifiers were used only to define the initial expressed background universe before identifier conversion. Because KEGG enrichment requires Entrez-based identifiers, both the background universe and the input DEG lists were mapped to Entrez Gene IDs using the org.Hs.eg.db Bioconductor annotation package (version 3.19.1; Bioconductor Project, Roswell Park Comprehensive Cancer Center, Buffalo, NY, USA) before GO and KEGG enrichment analysis. Thus, the final enrichment input and the final background universe supplied to clusterProfiler were Entrez ID-based. After Entrez mapping, the final explicit background universe contained 14,297 unique Entrez Gene IDs from 16,316 common post-filtered input identifiers. This reduction mainly reflected numeric identifiers, Ensembl identifiers present in the gene symbol field, and other unmapped or non-Entrez-mappable features, with consolidation to unique Entrez IDs after mapping. For the concordantly upregulated shared DEG list, 50 Entrez IDs were successfully mapped and retained within the explicit background universe from 52 input genes. For the concordantly downregulated shared DEG list, 123 Entrez IDs were successfully mapped from 210 input genes, of which 120 were retained within the explicit background universe. Unmapped genes and mapped genes outside the explicit background universe were recorded separately.

GO enrichment analysis was performed using enrichGO function from the ClusterProfiler version 4.12.6 (Bioconductor Project, Roswell Park Comprehensive Cancer Center, Buffalo, Erie County, New York 14263, USA) [92] with OrgDb = org.Hs.eg.db, keyType = “ENTREZID”, ont = “ALL”, and the explicit background universe supplied through the universe argument. The ont = “ALL” setting was used to evaluate Biological Process, Molecular Function, and Cellular Component terms. KEGG pathway enrichment analysis was performed using enrichKEGG function from the ClusterProfiler version 4.12.6 (Bioconductor Project, Roswell Park Comprehensive Cancer Center, Buffalo, Erie County, New York 14263, USA) with organism = “hsa”, keyType = “kegg”, and the same explicit background universe supplied through the universe argument. Multiple testing correction was performed using the Benjamini-Hochberg procedure, and enrichment terms were considered statistically significant at adjusted *p*-value < 0.05.

All enrichment outputs, mapped gene lists, unmapped gene lists, genes outside the explicit background universe, and background universe audit tables were saved for reproducibility. The audit files documented the number of post-filtered genes in each dataset, the number of genes retained in the common background universe before and after Entrez mapping, the number of input genes mapped to Entrez IDs, and the number of mapped input genes retained within the explicit background universe.

### 2.7 Pairwise Correlation Network Analysis

We performed an FDR-corrected pairwise coexpression network analysis [93] among the concordantly regulated shared DEGs identified between CLL and MM. This analysis was restricted to the concordantly regulated shared DEG set rather than all commonly expressed genes, to focus the network analysis on the core cross-disease expression signature that showed the same direction of regulation in both disease contexts.

To avoid directly integrating expression values from two independent datasets, coexpression networks were constructed separately for each dataset. The concordantly regulated shared DEG list contained 262 genes. Processed expression matrices from the primary analytical setting, corresponding to N = 1 filtering and pseudocount = 4, were used for both datasets. The CLL processed expression matrix was obtained from GSE70830, and the MM processed expression matrix was obtained from GSE196297. Genes were matched by gene symbol across the concordantly regulated shared DEG list and both processed expression matrices. Of the 262 concordantly regulated shared DEG symbols, 254 were present in both processed expression matrices and were retained for network construction.

Pairwise Pearson correlation coefficients were calculated independently within the CLL and MM datasets. Within each dataset, correlations were calculated between all unique gene pairs among the 254 retained genes. Self-correlations and duplicate undirected gene pairs were excluded. This produced 32,131 unique gene pairs tested separately in each dataset. For each gene pair, a two-sided correlation *p*-value was calculated from the Pearson correlation coefficient [94] using the t-statistic transformation:

$$t=\frac{r\sqrt{n-2}}{\sqrt{1-r^{2}}}$$

where r is the Pearson correlation coefficient, and n is the number of samples within the corresponding dataset. Thus, n = 15 was used for the CLL dataset, and n = 11 was used for the MM dataset. The resulting *p*-values were adjusted separately within each dataset across all 32,131 tested gene pairs using the Benjamini-Hochberg false discovery rate correction.

Within each dataset, gene pairs were retained as dataset-specific coexpression edges only if they met both an effect size threshold and a multiple testing threshold: an absolute Pearson correlation coefficient |r| ≥ 0.7 and an FDR-adjusted *p*-value < 0.05 [95]. The |r| ≥ 0.7 threshold was used to focus on strong coexpression relationships, while the FDR criterion controlled for multiple testing [96] across all pairwise correlations within each dataset.

To define the final cross-context coexpression network, the retained CLL and MM edge lists were intersected by gene-pair identity. Replicated coexpression edges were defined as gene pairs that passed the |r| ≥ 0.7 and FDR-adjusted *p*-value < 0.05 criteria independently in both datasets. To further reduce the risk of discordant or context-specific correlations being interpreted as shared coexpression, only replicated edges with the same correlation sign in both datasets were retained in the final network. Positive and negative replicated edges were recorded separately to distinguish concordant and inverse coexpression patterns.

Network-level summaries were generated by recording the number of concordantly regulated shared DEG symbols in the input list, the number of genes retained in both expression matrices, the number of unique gene pairs tested within each dataset, the number of edges retained within each dataset, the number of overlapping edges passing thresholds in both datasets, and the number of replicated same-sign edges. Hub genes were identified from the final replicated same-sign network using degree centrality, defined as the number of retained replicated edges connected to each gene. Betweenness and closeness centralities were also calculated as additional network descriptors. This analysis was interpreted as an exploratory, replicated coexpression analysis rather than as evidence of causal regulatory interactions.

### 2.8 Degree-Ranked Hub Gene Identification and Cutoff Sensitivity Analysis

Hub gene identification was performed using the final replicated same-sign coexpression network generated from the within-dataset CLL and MM edge-intersection workflow described above. In this network, nodes represented concordantly regulated shared DEG symbols retained in both processed expression matrices, and edges represented gene pairs that satisfied the predefined coexpression criteria independently in both datasets and showed the same correlation sign in the CLL and MM networks. Therefore, hub identification was performed only after within-dataset Pearson correlation testing, Benjamini-Hochberg FDR correction, cross-dataset edge intersection, and same-sign edge filtering had been completed.

The final replicated same-sign edge list was imported into R and analyzed as an undirected graph because pairwise coexpression relationships do not define regulatory directionality. Before graph construction, missing gene identifiers, empty gene identifiers, self-edges, and duplicate undirected edges were removed. Gene-pair identities were standardized by sorting the two gene names within each edge, so that reciprocal gene pairs were treated as the same undirected edge. The graph was then constructed using the igraph package version 2.1.4 [97] (Comprehensive R Archive Network/R Foundation for Statistical Computing; catalog number not applicable; Vienna, Austria) in R.

Hub genes were ranked primarily by degree centrality. Degree centrality was defined as the number of retained replicated same-sign edges connected to a given gene. This metric was selected because it directly quantifies local connectivity within the final replicated coexpression network and identifies genes connected to many other genes within the concordantly regulated shared DEG signature. All genes represented in the final network were ranked in descending order of degree. When genes had identical degree values, betweenness centrality, closeness centrality, and gene name were used as secondary ordering variables to generate a deterministic ranking.

Betweenness and closeness centralities were also calculated as complementary graph descriptors. Betweenness centrality was used to estimate the extent to which a node lies on shortest paths between other nodes, providing an additional measure of potential network bridging position. Closeness centrality was used to estimate how close a node is to all other nodes in the network, based on shortest-path distances. Degree centrality remained the primary hub-ranking metric because the objective of this analysis was to identify highly connected nodes within the replicated coexpression network rather than to infer causal regulation, information flow, or directed regulatory hierarchy.

To avoid relying on a single arbitrary cutoff to define hub genes, a cutoff sensitivity analysis was performed. The complete ranked gene list was evaluated using multiple hub-selection thresholds: the top 0.5%, 1%, 5%, and 10% of ranked genes, as well as fixed-count groups containing the top 10 and 20 genes. For each cutoff, the number of selected genes, the minimum and maximum degree values among selected genes, and the identities of selected genes were recorded. A membership table was also generated to document whether each ranked gene was retained under each hub definition. This analysis was used to evaluate whether highly ranked genes were consistently prioritized across strict and more permissive hub-selection schemes.

To evaluate whether the highest-degree nodes reflected stable network anchors rather than artifacts of dense random connectivity, three additional hub-stability checks were performed with a fixed random seed to ensure reproducibility.

First, an edge-count-matched random dense network comparison was performed. Random undirected networks were generated with the same number of nodes and the same number of edges as the observed replicated same-sign network. In each random network, edges were sampled uniformly without replacement from all possible undirected gene pairs. This procedure preserved the overall node count and edge burden of the observed network but randomized the connectivity pattern. The degree sequence was not preserved because the purpose of this test was to determine whether the observed hub degree structure exceeded what would be expected from a dense random graph with the same edge burden. For each random network, the maximum degree, the mean degree of the top 10 ranked nodes, and the mean degree of the top 20 ranked nodes were recorded. This procedure was repeated 5000 times. Empirical *p*-values were calculated using a one-count correction as the proportion of random values greater than or equal to the observed value: empirical *p* = (number of random values ≥ observed value + 1)/(number of random networks + 1). This check was designed to assess whether the observed highest-degree hubs were stronger than expected under dense random edge placement.

Second, an edge subsampling stability analysis was performed. In each subsampling iteration, 80% of the retained replicated same-sign edges were randomly selected, and the graph was reconstructed using the same node set. Degree centrality was recalculated for the subsampled graph, and genes were re-ranked by degree. The originally identified top-ranked genes were then evaluated for their retention within the top 10 and top 20 ranked positions across repeated subsampling iterations. This procedure was repeated 1000 times. The retention frequency of each originally top-ranked gene was recorded as the fraction of subsampling iterations in which that gene remained within the top 10 or top 20 ranked nodes. This analysis evaluated whether the hub ranking was robust to partial edge removal and whether the highest-degree genes remained highly ranked when the network was perturbed.

Third, a stricter edge-threshold sensitivity analysis was performed using the within-dataset CLL- and MM-retained edge lists. Dataset-specific edge lists were re-filtered using increasingly stringent correlation and FDR criteria. The evaluated settings included the primary threshold of absolute Pearson correlation coefficient |r| ≥ 0.70 with FDR-adjusted *p*-value < 0.05, stronger correlation thresholds of |r| ≥ 0.75 and |r| ≥ 0.80 with FDR-adjusted *p*-value < 0.05, and a stricter multiple-testing threshold of |r| ≥ 0.70 with FDR-adjusted *p*-value < 0.01. For each threshold setting, CLL and MM edge lists were filtered independently, intersected by gene-pair identity, and restricted to edges with the same correlation sign in both datasets. A new undirected network was then reconstructed for each threshold setting, and degree centrality ranking was repeated. The overlap of the resulting top 10 and top 20 ranked genes with the corresponding primary hub lists was recorded. This analysis evaluated whether hub prioritization was sensitive to the selected edge-retention threshold.

All hub-related outputs were saved for reproducibility, including the full degree-ranked centrality table, cutoff sensitivity summary, cutoff membership table, random dense network comparison summary, edge subsampling stability table, stricter threshold sensitivity summary, and the main manuscript hub table. The hub analysis was interpreted as an exploratory network prioritization procedure. Hub genes were therefore considered highly connected nodes within the replicated same-sign coexpression network and were not interpreted as confirmed causal regulators, experimentally validated disease drivers, or direct therapeutic targets.

### 2.9 Transcription Factor Annotation and Expression-Matched TF Overlap Analysis

#### 2.9.1 Input Scope, Concordant DEG Set, and TF Reference Definitions

The TF analysis input was explicitly defined to maintain a consistent gene flow across the revised analyses. The analysis was restricted to the current concordantly regulated shared DEG set, which contained 262 genes showing the same direction of differential expression in CLL and MM. Among these genes, 52 were upregulated in both diseases, and 210 were downregulated in both diseases. Discordantly regulated shared DEGs were not included in the revised TF annotation workflow because the objective of this analysis was to evaluate TF annotations within the shared concordant transcriptomic signature rather than the broader shared DEG set.

TF annotation was performed using complementary structured and curated resources. Strict TF annotation was defined using the Gene Ontology Molecular Function term “DNA-binding transcription factor activity” (GO:0003700) together with its offspring terms. A broader transcription regulator category was defined separately using the Gene Ontology Molecular Function term “transcription regulator activity” (GO:0140110) and its offspring terms. In parallel, genes were compared against the HumanTFs/Lambert-curated human TF catalog version 1.01 (HumanTFs/Lambert; Centre for Computational Biology and Bioinformatics, University of Toronto; catalog number not applicable; Toronto, Ontario, Canada), which contained 1639 TF symbols. For each gene, annotation status was recorded separately for strict GO TF annotation, broad GO transcription regulator annotation, HumanTFs/Lambert membership, and the combined strict GO or HumanTFs definition. This structure allowed TF candidates to be identified while preserving the distinction between direct TF annotation, broader transcription regulatory annotation, and genes without TF annotation support.

#### 2.9.2 TF Annotation of Degree-Ranked Genes from the FDR-Corrected Coexpression Network

Degree-ranked genes from the final FDR-corrected coexpression network were screened to determine whether the highest connectivity nodes had independent TF annotation support. The ranked network gene table contained 251 genes retained in the final replicated same-sign coexpression network. Because network degree reflects coexpression connectivity rather than regulatory function, this analysis was designed as an annotation screen rather than as evidence of TF activity or causal regulation.

Each degree-ranked gene was annotated using the same TF reference definitions described above, including strict GO TF annotation, broad GO transcription regulator annotation, HumanTFs/Lambert membership, and the combined strict GO or HumanTFs definition. TF annotation status was evaluated across predefined hub sensitivity cutoffs, including the top 0.5%, 1%, 5%, and 10% degree-ranked genes, as well as the top 10- and top 20-ranked genes. The hub cutoff membership table was linked to the final ranked gene table so that TF annotation could be interpreted under both proportional and fixed-count hub definitions.

To avoid overinterpreting highly connected genes as regulators, each ranked gene was assigned a conservative interpretation category. Genes with strict GO TF annotation or HumanTFs/Lambert membership were classified as annotated TF hub candidates, whereas genes with only broad transcription regulator annotation were kept as broad regulator candidates rather than strict TFs. Genes without TF or broad transcription regulator annotations were interpreted only as network connectivity hubs. Hub reliability information from the random dense network anchor check and edge subsampling stability analysis was retained as network robustness context, but it was not treated as evidence of TF activity.

#### 2.9.3 TF Annotation of Concordantly Regulated Shared DEGs

The concordantly regulated shared DEG set was also evaluated independently of the network ranking analysis to identify TF-annotated genes within the shared transcriptomic signature. This analysis used the same 262 concordantly regulated shared DEGs defined above, while preserving the direction of differential expression for each gene in both disease comparisons. Genes with positive log_2_ fold change values in both CLL and MM were classified as upregulated in both diseases, whereas genes with negative log_2_ fold change values in both comparisons were classified as downregulated in both diseases.

Each concordantly regulated shared DEG was annotated using the same TF reference framework applied to the degree-ranked network genes. Annotation categories included strict GO TF annotation, broad GO transcription regulator annotation, HumanTFs/Lambert membership, the combined strict GO or HumanTFs definition, and the combined TF or broad transcription regulator definition. Genes satisfying either a strict GO TF annotation or membership in HumanTFs/Lambert were retained as annotated TF candidates within the concordant shared DEG signature.

This DEG-level TF screen was intentionally kept separate from the hub annotation screen because differential expression and network connectivity represent distinct biological and analytical properties. A gene could be a concordantly regulated shared DEG without being a high-degree network hub, and a highly connected network gene could lack TF annotation. No borderline TF rescue screen or joint multi-dataset testing was performed; the analysis remained restricted to genes satisfying the final concordant shared DEG criteria in both disease comparisons.

#### 2.9.4 Expression Matched Permutation Assessment of TF Annotation Overlap

To determine whether TF annotations were overrepresented among the concordantly regulated shared DEG signature, a permutation-based TF overlap assessment was performed using an expression-matched null model. The observed gene set was defined as the background-compatible subset of the 262 concordantly regulated shared DEGs, corresponding to 170 genes retained in the explicitly expressed background universe. The background universe contained 14,296 gene symbols with expression values available in both processed expression matrices. For each background gene, a combined expression abundance metric was calculated from the mean expression across the processed CLL and MM matrices and used for expression matching.

The primary permutation analysis used 20 expression abundance bins and 100,000 random permutations with a fixed random seed. Within each permutation, random gene sets were sampled from the expressed background while preserving the expression bin structure of the observed gene set, thereby controlling for potential differences in expression abundance distributions between TFs and other genes. The observed TF count was compared with the resulting expression-matched null distribution for each TF definition, including strict GO TF annotation, HumanTFs/Lambert membership, the combined strict GO or HumanTFs definition, broad GO transcription regulator annotation, and the combined TF or broad transcription regulator definition.

Empirical enrichment and depletion *p*-values were calculated from the expression-matched null distribution using one-count correction. Expected TF counts, fold over expected values, null distribution standard deviations, and z scores were also calculated. To evaluate robustness to binning choices, the expression-matched permutation analysis was repeated with 10 and 30 expression bins. Uniform random permutation and hypergeometric tests were retained only as secondary analytical references because these approaches do not directly control for expression abundance matching. TF overlap results were interpreted as evidence of annotation enrichment rather than of TF activity or causal regulation.

## 3 Results

### 3.1 Mean Variance Plots after VST Transformation

Supplementary Fig. S1 presents the mean versus standard deviation for the CLL and MM datasets after variance-stabilizing transformation. These plots were used to evaluate whether the transformed expression matrices showed reduced dependence between average expression magnitude and gene-level variability. In RNA sequencing data, lowly and moderately expressed genes commonly exhibit stronger mean-variance relationships, and variance stabilization aims to reduce this dependence for exploratory sample-level analyses such as PCA and visualization.

In both datasets, most genes were concentrated at lower-to-intermediate expression levels, consistent with the expected distribution of bulk RNA sequencing data in which many genes are expressed at relatively low abundance. The fitted trendlines indicated that the relationship between mean expression and standard deviation was reduced after VST across much of the expression range. The CLL dataset showed a relatively smooth variance profile, with moderate variability among lower- to intermediate-expression values and reduced variability across much of the higher-expression range. The MM dataset showed a broader spread and a modest residual increase in variability among higher-expression genes, which may reflect dataset-specific biological heterogeneity, sample composition, or residual expression level-dependent variability. Overall, these plots support the use of VST-transformed matrices for exploratory sample-level assessment, including PCA-based visualization. However, the primary differential expression analyses were performed using raw count data within DESeq2, as described in the Methods section.

### 3.2 Scree Plots for CLL and MM

Supplementary Fig. S2 presents scree plots for the CLL and MM datasets after dimensionality reduction of the transformed expression matrices. These plots were used to assess the distribution of variance across principal components and to determine whether the main structure of each dataset was concentrated within the leading components. In both datasets, the first principal component explained the largest proportion of variance, accounting for approximately 40% in the CLL dataset and approximately 34% in the MM dataset. The second principal component contributed additional variance, and together the first two components explained more than half of the total variance in each dataset.

The scree plots showed a marked decline in incremental explained variance after the leading components, indicating that most of the dominant sample-level structure was captured by the early principal components. To further support component retention, Horn’s Parallel Analysis was performed to compare the observed variance explained by each principal component with the variance expected under random sampling. In both datasets, the first three principal components exceeded the chance-adjusted variance threshold, supporting the interpretation that the dominant structure of the transformed expression matrices was concentrated within the first three components. Indeed, these findings indicate that the CLL and MM datasets each contained a structured variance pattern rather than a diffuse distribution of variance across many components. This supported the use of the leading principal components for subsequent exploratory visualization and sample-level assessment. Biological interpretation of these components was then based on their relationship to disease and control sample separation in the corresponding PCA analyses.

### 3.3 PCA Analysis of CLL and MM Datasets

Supplementary Fig. S3 illustrates the principal component analysis of the CLL and MM datasets based on the VST-transformed expression matrices. In the CLL dataset, the PCA plot showed clear separation between CLL samples and control CD19-positive B cell samples along PC1, which accounted for 40.81% of the total variance. This pattern suggests that a major source of transcriptomic variation in the CLL dataset was associated with disease versus control status.

Similarly, the MM dataset showed a separation between MM-associated and control MSC samples, although it was less pronounced than in the CLL dataset. In the MM PCA plot, PC1 accounted for 34.13% of the variance, while PC2 accounted for an additional 20.01%. The broader dispersion among MM-associated MSC samples may reflect greater biological heterogeneity, MSC-related sample composition, and the smaller cohort consisting of six MM-associated MSC samples and five control MSC samples. In fact, the PCA results support the presence of disease-associated sample-level transcriptomic structure in both datasets relative to their respective control groups. These findings are consistent with the scree plot results, which showed that the leading principal components captured a substantial proportion of the total variance in both CLL and MM.

### 3.4 DEG Counts, Directionality, and Surrogate Variable Sensitivity Analysis

**Figure 1:**
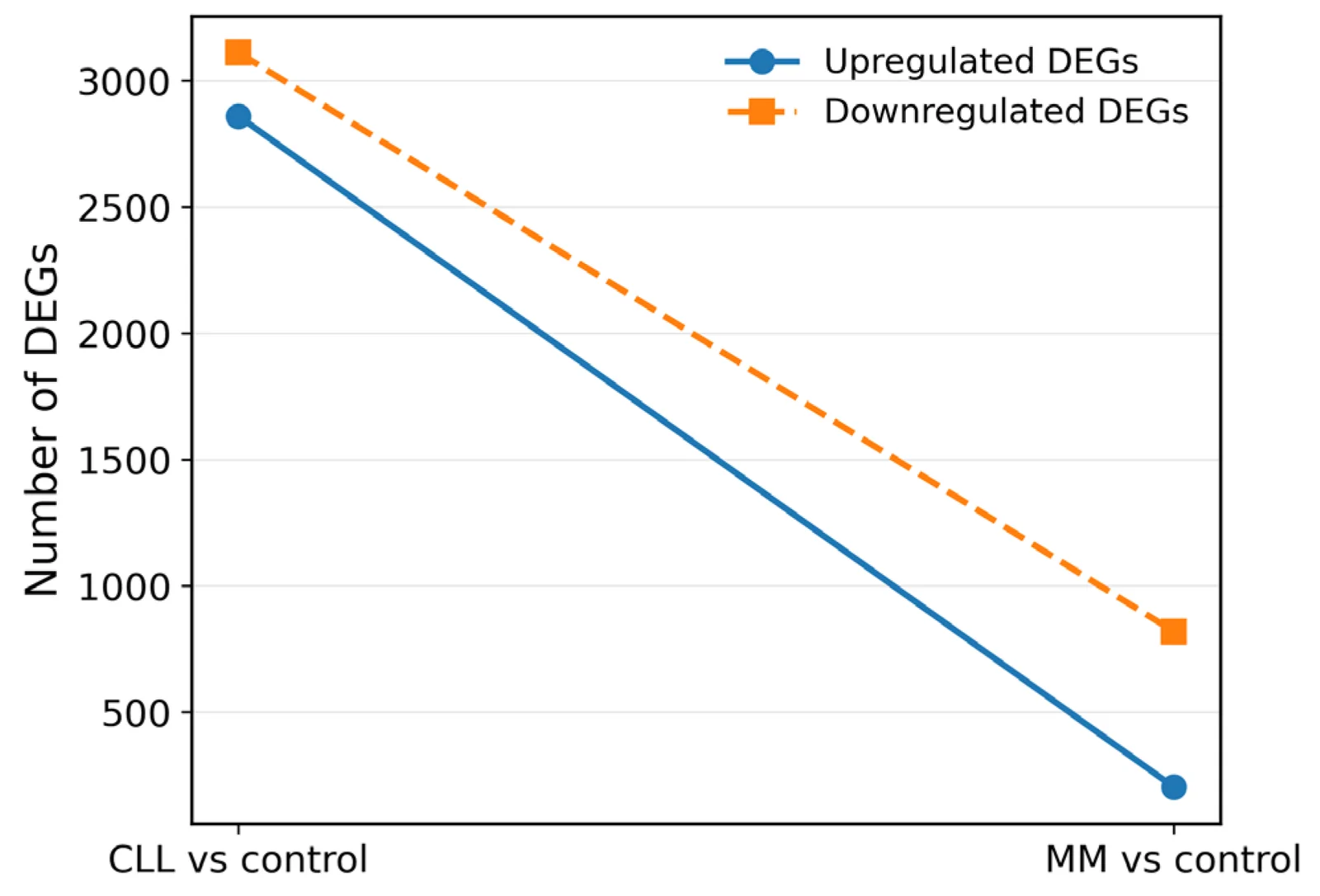
DEG Line Plot for CLL and MM. This line plot summarizes the numbers of significantly upregulated and downregulated genes identified in the CLL and MM datasets using the manuscript threshold of adjusted *p*-value < 0.05 and absolute log_2_ fold change > 1. In total, 5965 DEGs were identified in the CLL dataset, whereas 1021 DEGs were identified in the MM dataset. The CLL comparison showed a larger detected DEG burden than the MM comparison, consistent with a broader detected disease-associated transcriptional difference in CLL relative to its matched control CD19-positive B cell samples. In contrast, the MM-associated MSC comparison showed a smaller DEG burden, with downregulated genes representing a larger proportion of the identified DEGs.

The difference in DEG burden between the two datasets in Fig. 1 likely reflects multiple factors, including disease-specific biology, cohort size, sample composition, and cell type context. The CLL dataset was based on CD19-positive B cells and included a larger cohort, potentially increasing the ability to detect disease-associated transcriptional differences. In contrast, the MM dataset was based on bone marrow-derived MSCs and included six MM-associated samples and five control MSC samples. MSCs are biologically heterogeneous and strongly influenced by their microenvironment, which may contribute to broader within-group variability and a more conservative DEG profile. Therefore, the DEG counts should be interpreted as dataset-specific detected transcriptional patterns rather than as a direct quantitative comparison of disease severity, biological activity, or global transcriptional disruption between CLL and MM.

To further evaluate the robustness of the differential expression results, we performed an exploratory surrogate variable adjustment sensitivity analysis using the primary N = 1 filtering setting with pseudocount = 4. In the CLL dataset, the expression signature remained highly stable after surrogate variable adjustment. With one surrogate variable, the DEG Jaccard similarity was 0.9558, and Pearson and Spearman correlations of log_2_ fold change values were 0.999991 and 0.999985, respectively. With two surrogate variables, the DEG Jaccard similarity was 0.8941, while Pearson and Spearman log_2_ fold change correlations remained very high at 0.999315 and 0.999097, respectively.

In the MM dataset, threshold-based DEG classification was more sensitive to surrogate variable adjustment, consistent with the smaller cohort size and greater biological heterogeneity of MSC-derived samples. However, the overall effect size structure remained strongly concordant. With one surrogate variable, Pearson and Spearman log_2_ fold-change correlations were 0.997943 and 0.996874, respectively. With two surrogate variables, the corresponding correlations were 0.951374 and 0.939908. Directional concordance was also high, particularly among genes with larger effect sizes. Together, these findings indicate that the main direction and magnitude of disease-associated expression changes were largely preserved after exploratory surrogate variable adjustment, while threshold-based DEG calls in the smaller MM dataset should be interpreted with appropriate caution.

### 3.5 DEG Overlap in CLL and MM

**Figure 2:**
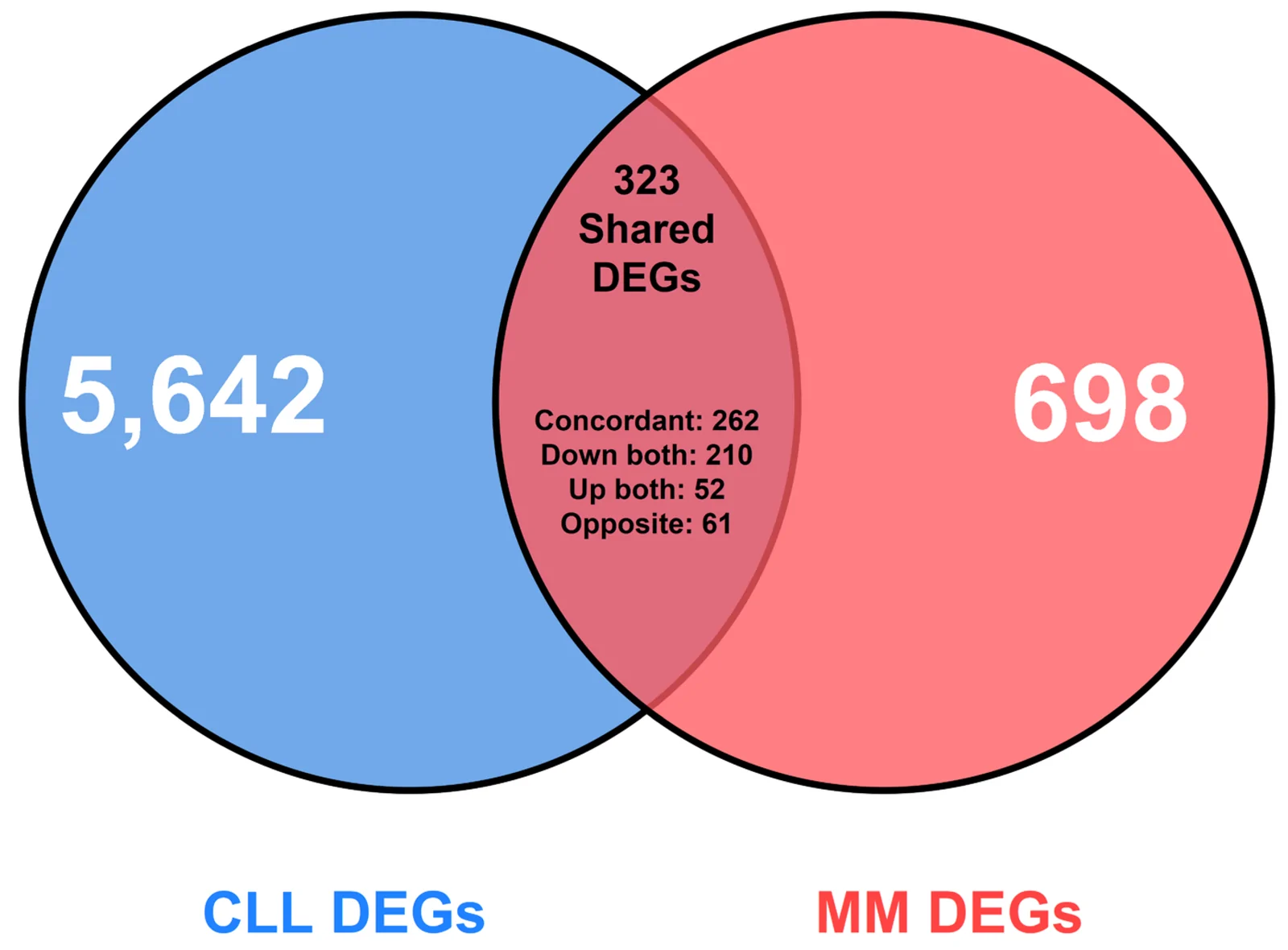
DEG Overlap between CLL and MM. This Venn diagram shows the overlap between significant DEGs identified in the CLL and MM datasets using adjusted *p*-value < 0.05 and absolute log_2_ fold change > 1. The CLL comparison included 5965 DEGs, with 5642 CLL-specific DEGs. The MM comparison included 1021 DEGs, with 698 MM-specific DEGs. A total of 323 DEGs were shared between the two comparisons, including 262 concordantly regulated genes, 210 downregulated in both diseases, 52 upregulated in both diseases, and 61 genes with opposite directions of regulation.

Fig. 2 shows the overlap between significant DEGs identified in the CLL and MM datasets using the final DEG criteria. In the CLL dataset, 5965 DEGs were identified, of which 5642 were specific to the CLL comparison. In the MM dataset, 1021 DEGs were identified, of which 698 were specific to the MM comparison. A total of 323 significant DEGs were shared between the CLL and MM comparisons.

Among these 323 shared significant DEGs, 262 showed concordant direction of regulation in both diseases. Specifically, 210 genes were downregulated in both CLL and MM, whereas 52 genes were upregulated in both diseases. The remaining 61 shared DEGs showed opposite regulatory directions between the two disease comparisons. The predominance of concordantly downregulated shared DEGs suggests that a subset of disease-associated transcriptomic signals may be commonly reduced in both CLL and MM, potentially reflecting shared alterations in differentiation-related processes, immune regulatory activity [98,99,100,101,102,103,104], stromal signaling [105,106], or disease-associated transcriptional control [107,108,109]. However, the CLL and MM datasets were derived from different cell types, CD19-positive B cells and bone marrow-derived MSCs, respectively. Thus, these shared DEGs should be interpreted as convergent disease-associated transcriptional signals rather than as evidence of identical molecular mechanisms.

The shared DEGs presented in Fig. 2 were interpreted as convergent disease-associated transcriptional signals across the two disease contexts rather than as evidence of identical molecular mechanisms [110].

### 3.6 Cross-Disease DEG Log_2_FC Concordance Analysis

To determine whether the concordantly regulated shared DEGs showed similarity beyond the same direction of regulation, we evaluated the correlation of log_2_ fold change values between CLL and MM across the 262 shared DEGs regulated in the same direction in both disease comparisons. As described above, these 262 concordantly regulated shared DEGs included 52 genes upregulated in both CLL and MM and 210 genes downregulated in both diseases. The remaining 61 shared significant DEGs showed opposite directions of regulation and were therefore excluded from the same-direction concordance analysis.

**Figure 3:**
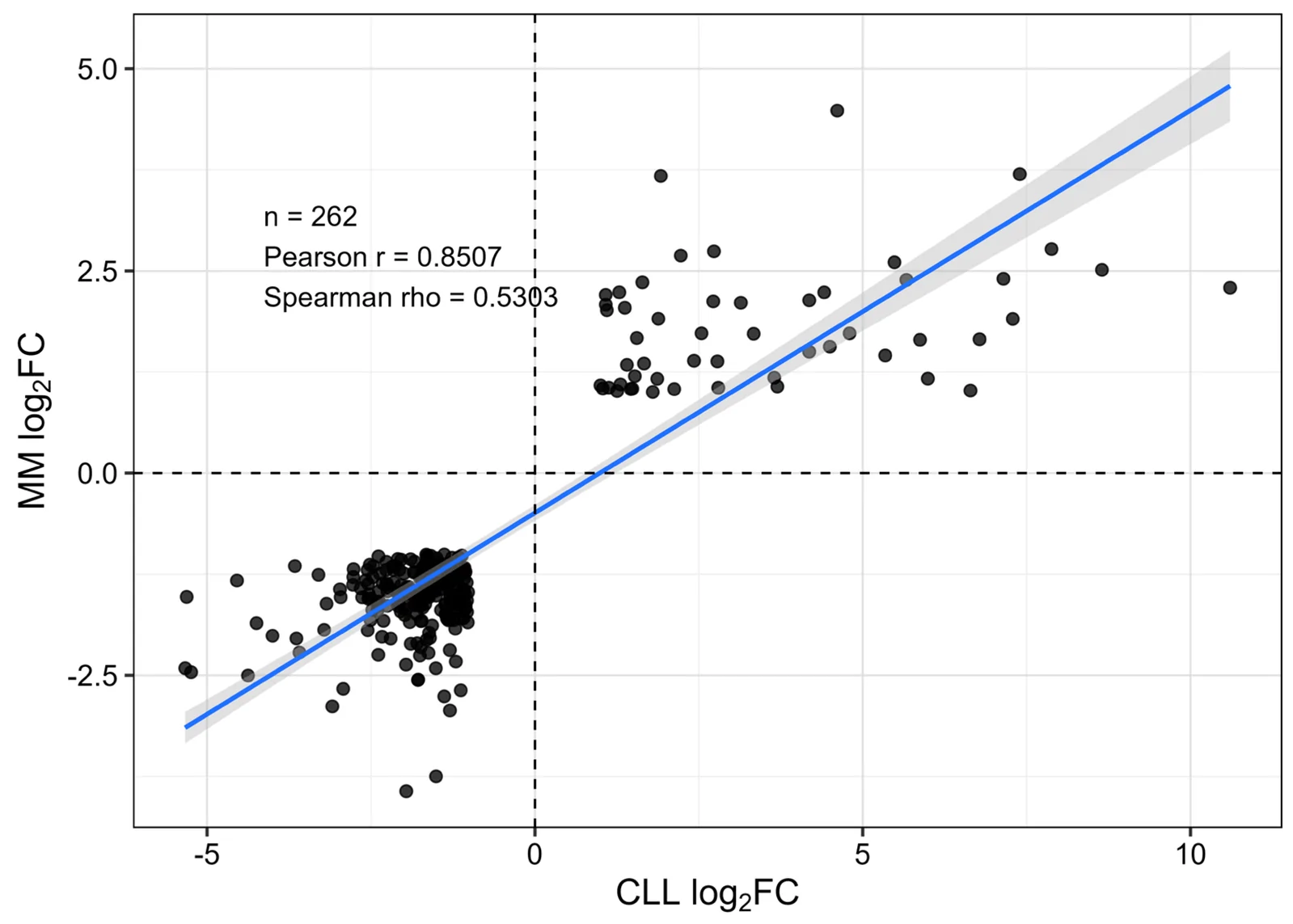
Log_2_ Fold Change Concordance Across Concordantly Regulated Shared DEGs between CLL and MM. This scatter plot shows the relationship between CLL and MM log_2_ fold change values across the 262 shared DEGs that were regulated in the same direction in both disease comparisons. Each point represents one concordantly regulated shared DEG. The *x*-axis shows the CLL versus control log_2_ fold change, and the *y*-axis shows the MM versus control log_2_ fold change. Dashed horizontal and vertical lines indicate log_2_ fold change equal to zero. The analyzed gene set included 52 genes upregulated in both diseases and 210 genes downregulated in both diseases. The regression line and confidence band show the positive association between CLL and MM log_2_ fold change values. Pearson correlation showed strong positive concordance between CLL and MM effect sizes across these genes (r = 0.8507), while Spearman rank correlation was also positive (ρ = 0.5303). These results also support that the concordantly regulated shared DEG subset reflects both directional agreement and similarity in overall log_2_ fold change structure between CLL and MM.

Among the 262 concordantly regulated shared DEGs, CLL and MM log_2_ fold change values exhibited a strong positive Pearson correlation (r = 0.8507, *p* = 1.45 × 10^−74^). Spearman’s rank correlation was also positive and statistically significant (ρ = 0.5303, *p* = 2.09 × 10^−20^). These findings indicate that the concordant shared DEG subset reflects not only agreement in the direction of regulation but also similarity in the overall structure of effect sizes between the two disease comparisons (Fig. 3).

Although the magnitudes of fold change were not identical between CLL and MM, the observed positive correlations support the presence of a shared transcriptomic pattern among the concordantly regulated overlapping DEGs. The median absolute log_2_ fold change was 1.762 in CLL and 1.499 in MM, with a median absolute log_2_ fold change difference of 0.508 across the concordantly regulated shared DEG set. These results suggest that this shared DEG subset may reflect a biologically concordant expression signature while preserving disease-specific differences in the magnitude of transcriptional dysregulation.

### 3.7 Cross-Disease Pathway Enrichment Analysis

To assess pathway-level convergence between CLL and MM, we performed GO and KEGG enrichment analyses on the concordantly upregulated and downregulated shared DEG sets, using an explicitly defined expressed-gene background universe. The background universe was constructed from the intersection of post-filtered genes retained in the primary processed expression matrices used for the study, corresponding to the N = 1 filtering setting and pseudocount = 4. The CLL processed matrix contained 20,117 post-filtered genes, and the MM processed matrix contained 18,700 post-filtered genes. Their intersection contained 16,316 common gene symbols, which were subsequently mapped to 14,297 unique Entrez Gene IDs and used as the explicit enrichment background.

Among the concordantly regulated shared DEGs, 52 genes were upregulated in both CLL and MM, of which 50 mapped Entrez IDs were retained within the explicit background universe. In contrast, 210 genes were downregulated in both diseases, of which 123 mapped to Entrez IDs and 120 were retained within the explicit universe. GO and KEGG enrichment analyses were then performed separately for the concordantly upregulated and concordantly downregulated shared DEG sets.

After applying the explicit post-filtered background universe, no GO or KEGG enrichment terms remained statistically significant after Benjamini-Hochberg multiple testing correction. This was observed for both the concordantly upregulated and concordantly downregulated shared DEG sets across GO Biological Process, GO Molecular Function, GO Cellular Component, and KEGG pathway analyses. These findings indicate that the concordantly shared DEG signature did not yield robust pathway-level enrichment when evaluated against a dataset-specific expressed gene background. Because no GO or KEGG terms remained significant under this conservative background definition, pathway enrichment results were not used as primary evidence for shared disease biology. Instead, the shared transcriptomic relationship between CLL and MM was supported primarily by gene-level evidence, including DEG overlap, concordant direction of regulation among the 262 shared DEGs, and significant correlation in log_2_ fold change values across this set of concordantly regulated DEGs.

An additional enrichment analysis was performed using the combined pool of concordantly regulated shared DEGs, including both concordantly upregulated and downregulated DEGs. After Entrez mapping, 173 unique Entrez IDs were identified from the combined concordant DEG pool, of which 170 were retained within the explicitly expressed gene background universe. Using the same explicit background universe and Benjamini-Hochberg multiple testing correction, no GO or KEGG terms remained statistically significant for the combined concordant DEG pool. Therefore, the combined analysis did not change the main interpretation that the shared CLL and MM transcriptomic signal was supported primarily at the gene level rather than by robust pathway-level overrepresentation.

The lack of GO or KEGG terms remaining significant after correction using the expressed gene background indicates that the shared CLL and MM DEG signature was not dominated by a broad generalized pathway-level enrichment (Supplementary Fig. S4). This result should be interpreted in the context of conventional overrepresentation analysis, which tests whether predefined functional categories are statistically enriched in an input gene list relative to a selected background gene universe [111,112]. Because enrichment results can be sensitive to the composition of the input DEG list, the selected background universe, and the structure of existing pathway annotations, a negative adjusted enrichment result does not necessarily imply the absence of a biological signal [111,113]. Rather, it may indicate that the common CLL and MM signal is more apparent at the gene level and is not fully captured by broad GO or KEGG category-level overrepresentation [112,113]. This interpretation is consistent with the concordant DEG patterns and annotated TF candidates identified in the present study. It also supports the need for higher resolution validation using larger cohorts, independent datasets, and single-cell transcriptomic approaches, which can better resolve cell-type-specific and microenvironment-associated transcriptional programs [114].

### 3.8 FDR-Corrected Pairwise Co-Expression Network Analysis

To evaluate coordinated expression patterns within the concordantly regulated shared DEG signature between CLL and MM, we performed an FDR-corrected pairwise coexpression network analysis in each dataset independently. The analysis was restricted to the 262 shared DEGs that showed the same direction of regulation in both disease comparisons, thereby focusing on the concordant cross-context DEG set identified in the overlap and directionality analyses.

Of the 262 concordantly regulated shared DEG symbols used as input, 254 were present in both N = 1, pseudocount = 4 processed expression matrices and were retained for network construction. Pairwise Pearson correlations were calculated separately within the GSE70830 CLL expression matrix and the GSE196297 MM expression matrix. Within each dataset, only unique undirected gene pairs were tested, and self-correlations were excluded. This resulted in 32,131 unique gene-pair comparisons, evaluated independently in each dataset.

For each gene pair, a two-sided Pearson correlation *p*-value was calculated within each dataset, followed by Benjamini-Hochberg FDR correction across all tested gene pairs in that dataset. Dataset-specific edges were retained only when they satisfied both predefined criteria: absolute Pearson correlation coefficient |r| ≥ 0.7 and FDR-adjusted *p*-value < 0.05. Using these criteria, 15,950 edges were retained in the CLL within the dataset network, and 18,404 edges were retained in the MM within the dataset network.

To identify coexpression relationships that were reproducible across both disease contexts, the retained CLL and MM edge lists were intersected by gene pair identity. Gene pairs were included in the final replicated coexpression network only if they met the correlation magnitude and FDR thresholds independently in both datasets and showed the same correlation sign in both datasets. This analysis identified 11,233 replicated same-sign edges involving 251 genes (Table 1).

The replicated same-sign network was used for downstream hub gene prioritization. Degree centrality was calculated as the number of replicated edges connected to each gene, and betweenness and closeness centralities were calculated as additional network descriptors. Because the analysis was based on retrospective public datasets with modest sample sizes and distinct cellular contexts, the resulting network was interpreted as exploratory coexpression evidence rather than as causal regulatory inference.

**Table 1:** Summary of FDR-corrected within-dataset pairwise coexpression network analysis.

Metric	Value
Input gene set	Concordantly regulated shared DEGs between CLL and MM
Concordantly regulated shared DEG symbols in input list	262
Shared DEG symbols retained in both expression matrices	254
CLL samples analyzed	15
MM samples analyzed	11
Unique gene pairs tested within each dataset	32,131
Correlation method	Pearson correlation
Correlation threshold	|r| ≥ 0.7
Multiple testing correction	Benjamini-Hochberg FDR correction applied separately within each dataset
FDR threshold	FDR < 0.05
CLL within-dataset retained edges	15,950
MM within-dataset retained edges	18,404
Overlapping same-sign edges passing thresholds in both datasets	11,233
Final replicated same-sign edges	11,233
Genes represented in the final replicated network	251
Final replicated same-sign edges among tested pairs	34.96%

Note: DEG, differentially expressed gene; FDR, false discovery rate. Pairwise coexpression networks were constructed independently within the CLL and MM datasets using the concordantly regulated shared DEG set. Edges were retained within each dataset only when gene pairs satisfied both |Pearson r| ≥ 0.7 and Benjamini-Hochberg FDR-adjusted *p*-value < 0.05. The final network retained only replicated same-sign edges observed in both datasets. Eight input DEG symbols were not retained because they were not present in both N = 1, pseudocount = 4 processed expression matrices after gene-symbol matching. Therefore, matched expression vectors were not available for these genes in both datasets for pairwise coexpression analysis.

### 3.9 Degree-Ranked Hub Gene Identification and Anchor Sensitivity Analysis

In this analysis, we ranked genes within the final replicated same-sign coexpression network to identify highly connected hub genes and evaluated the stability of these rankings across complementary sensitivity checks. Degree centrality was calculated for the genes represented in the final replicated same-sign coexpression network. This analysis was performed on the finalized replicated edge list generated after independent FDR correction and same-sign edge intersection across the CLL and MM datasets. The resulting network contained 251 nodes and 11,233 replicated edges, with a network density of 0.358 and a mean degree of 89.51. Before hub ranking, the replicated edge list was processed as unique undirected gene pairs, and the downstream input audit confirmed that the analysis used the finalized CLL and MM retained edge files containing 15,950 and 18,404 edges, respectively. Thus, the hub analysis was directly linked to the FDR-corrected network described above and was not performed on an intermediate or unfiltered edge set.

**Figure 4:**
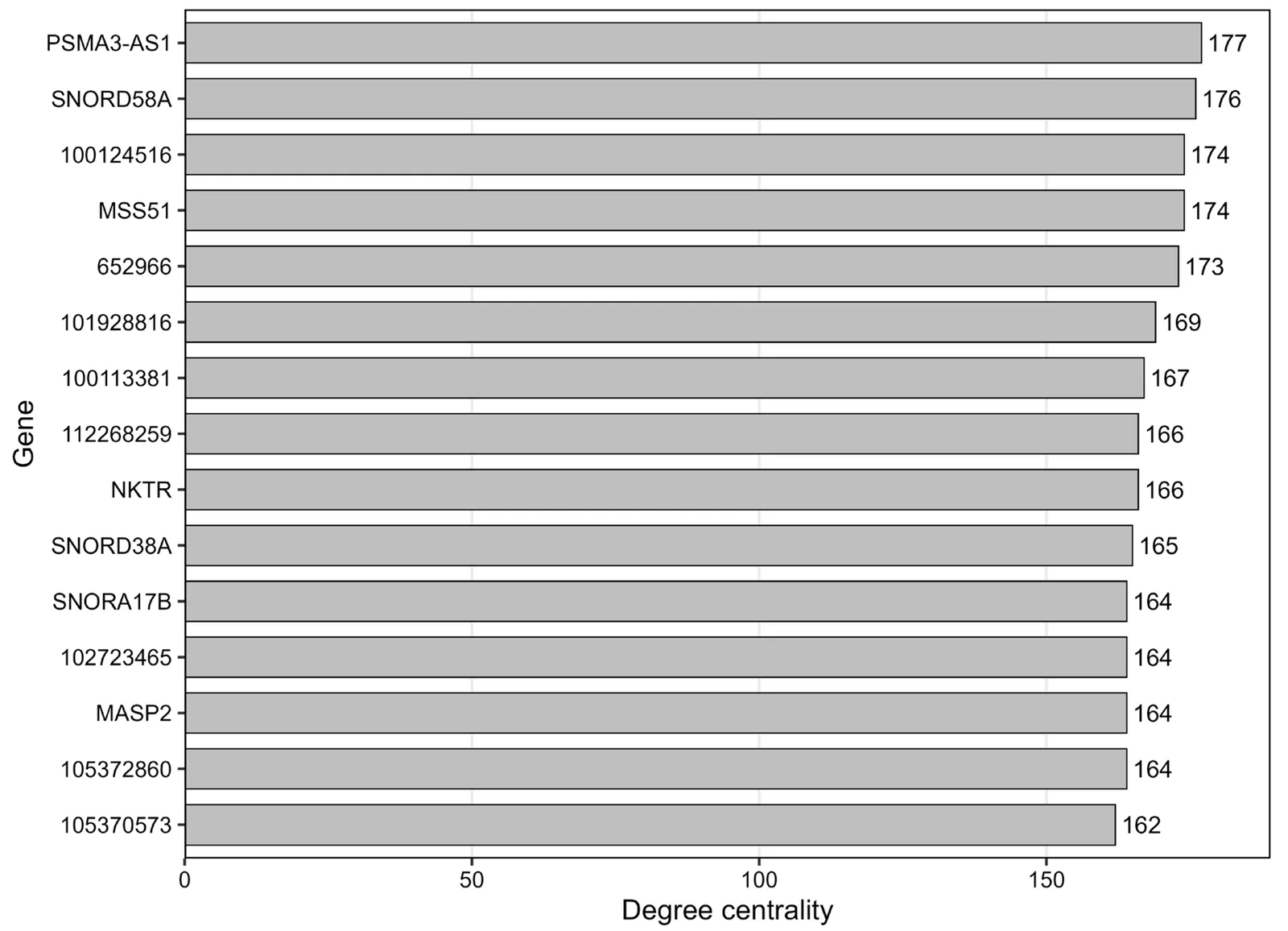
Top hub genes in the FDR-corrected replicated same-sign coexpression network of concordantly regulated shared DEGs. The bar plot shows the top 15 genes ranked by degree centrality within the final replicated same-sign coexpression network constructed from concordantly regulated shared DEGs between CLL and MM. The network contained 251 genes and 11,233 replicated edges. Degree represents the number of retained, FDR-corrected, replicated same-sign edges connected to each gene. Hub genes were interpreted as exploratory indicators of network connectivity rather than evidence of causal regulatory control.

The highest degree nodes showed connectivity values substantially above the network mean. The top-ranked hub gene was PSMA3-AS1, with a degree of 177, followed by SNORD58A with a degree of 176. The next-highest-ranked genes were 100124516 and MSS51, each with a degree of 174, followed by 652966, 101928816, 100113381, 112268259, NKTR, and SNORD38A. These genes formed the leading degree-ranked set in the replicated network and are summarized in Fig. 4 and Table 2. Because degree centrality represents the number of replicated FDR-corrected same-sign edges connected to each gene, these results identify the most highly connected nodes within the shared CLL and MM coexpression structure rather than genes inferred to have causal regulatory activity.

To evaluate whether hub designation depended on a single arbitrary cutoff, hub membership was assessed using percentile-based and fixed-count definitions. The top 0.5% cutoff retained two genes, PSMA3-AS1 and SNORD58A, with degree values of 177 and 176, respectively. The top 1% cutoff additionally retained 100124516, with degree values ranging from 174 to 177. Broader cutoffs retained larger but still high-degree gene sets: the top 5% cutoff retained 13 genes with degrees ranging from 164 to 177, whereas the top 10% cutoff retained 26 genes with degrees ranging from 154 to 177. Fixed count definitions showed similar behavior, with the top 10 genes spanning degree values of 165 to 177 and the top 20 genes spanning 158 to 177. This cutoff-sensitivity pattern indicated that the leading hub signal was concentrated in a small group of highly connected genes. Yet, it remained visible under broader hub definitions.

Because the final replicated network was dense, we further evaluated whether the observed hub structure exceeded that expected for a dense network with the same number of nodes and edges. Across 5000 edge-count-matched random networks, the observed maximum degree of 177 was substantially higher than the random mean maximum degree of 111.16 and exceeded the random 99th percentile of 120. The observed mean degree of the top 10 hubs was 170.70, compared with a random 95th percentile value of 107.80. Similarly, the observed mean degree of the top 20 hubs was 166.20, compared with a random 95th percentile value of 105.05. The empirical *p*-value was 0.0002 for the maximum degree, top 10 mean degree, and top 20 mean degree comparisons. These results support that the leading hubs were not explained by network density alone.

Edge subsampling was then used to assess the stability of hub ranking under partial edge perturbation. In 1000 subsampling iterations retaining 80% of edges per iteration, the top three genes, PSMA3-AS1, SNORD58A, and 100124516, remained in the top 10 in 90.3% to 93.7% of iterations and remained in the top 20 in 99.2% to 99.6% of iterations. The top five genes were retained within the top 20 in 98.5% to 99.6% of iterations, and all original top 10 genes showed top 20 retention frequencies of at least 84.2%. This stability analysis indicates that the leading hub assignments were not driven by a small number of individual edges and remained largely preserved after repeated random removal of a substantial fraction of network edges.

The hub ranking was also evaluated under stricter edge retention settings to test whether the leading nodes persisted when the replicated network was made more conservative. Under the primary threshold of |r| ≥ 0.70 and FDR < 0.05, the network contained 11,233 edges and 251 nodes. Increasing the correlation threshold to |r| ≥ 0.75 retained 7813 edges and 234 nodes, while |r| ≥ 0.80 retained 4680 edges and 215 nodes. Applying a stricter FDR threshold of FDR < 0.01 while retaining |r| ≥ 0.70 yielded 8049 edges and 238 nodes. Despite these reductions in network size, the original top three genes, PSMA3-AS1, SNORD58A, and 100124516, remained within the top 20 under all stricter settings. In addition, 16 to 17 of the original top 20 hubs were retained across the stricter networks, showing that the hub signal was not uniquely dependent on the primary correlation or FDR threshold.

**Table 2:** Degree-ranked hub genes in the FDR-corrected replicated same-sign coexpression network of concordantly regulated shared DEGs.

Gene	Rank	Degree	Betweenness	Closeness
PSMA3-AS1	1	177	0.0108	0.7278
SNORD58A	2	176	0.0125	0.7321
100124516	3	174	0.0131	0.7300
MSS51	4	174	0.0056	0.7069
652966	5	173	0.0055	0.7069
101928816	6	169	0.0144	0.7130
100113381	7	167	0.0044	0.6949
112268259	8	166	0.0090	0.6969
NKTR	9	166	0.0035	0.6796
SNORD38A	10	165	0.0088	0.7009
SNORA17B	11	164	0.0062	0.6852
102723465	12	164	0.0045	0.6891
MASP2	13	164	0.0042	0.6721
105372860	14	164	0.0041	0.6796
105370573	15	162	0.0061	0.6721

Note: DEG, differentially expressed gene; FDR, false discovery rate. The coexpression network was constructed from the concordantly regulated shared DEG set between CLL and MM. Hub genes were ranked by degree centrality in the final replicated same-sign network, which contained 251 genes and 11,233 replicated edges. Degree represents the number of retained, FDR-corrected, replicated same-sign network edges connected to each gene. Betweenness and closeness are provided as additional graph centrality descriptors. Hub genes were interpreted as exploratory indicators of network connectivity rather than as causal regulators.

The degree-ranked hub analysis identified a reproducible set of high-connectivity nodes within the final replicated same-sign coexpression network. The convergence of multiple checks, including cutoff sensitivity, dense random network comparison, edge subsampling stability, and stricter threshold sensitivity, supports the robustness of the leading hub rankings. These genes were therefore interpreted as exploratory network anchors within the shared CLL and MM coexpression structure while avoiding causal regulatory interpretation from coexpression data alone.

### 3.10 Transcription Factor Annotation and Expression Matched TF Overlap Assessment

To evaluate whether the concordantly regulated shared CLL and MM transcriptomic signature contained a transcription factor component, TF annotation was applied to the final set of concordant shared DEG used for the revised analysis. This set contained 262 concordantly regulated shared DEGs, including 52 genes upregulated in both diseases and 210 genes downregulated in both diseases. No discordant genes were retained in this TF analysis input.

**Figure 5:**
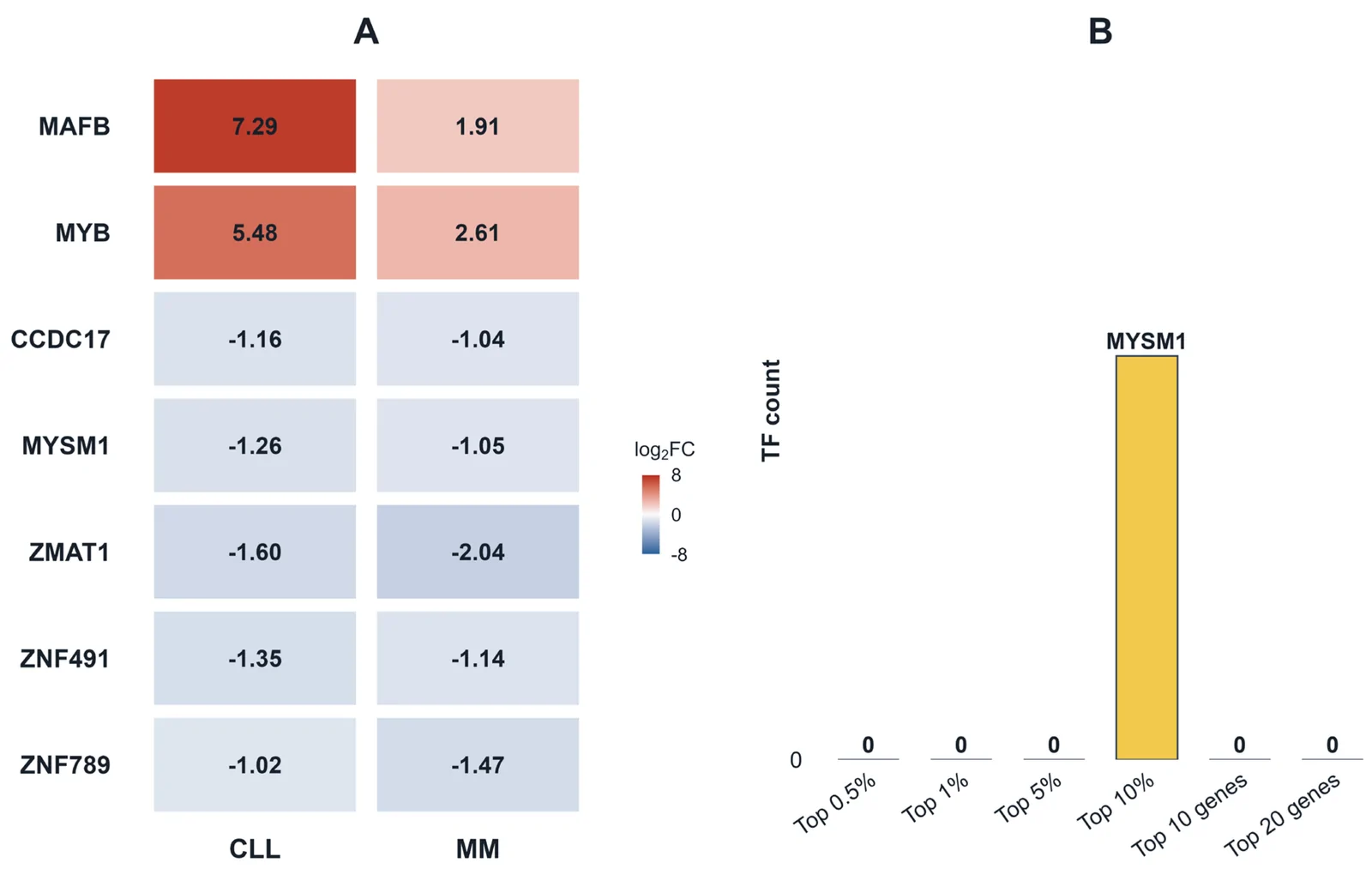
TF annotation of the concordantly regulated shared CLL and MM DEG signature. (**A**) Heatmap showing the log_2_ fold changes of the seven TF-annotated concordant shared DEGs in CLL and MM. MAFB and MYB were upregulated in both diseases, whereas CCDC17, MYSM1, ZMAT1, ZNF491, and ZNF789 were downregulated in both diseases. (**B**) TF annotation across degree-ranked hub cutoffs in the FDR-corrected coexpression network. No TFs were detected among the top 0.5%, top 1%, top 5%, top 10 genes, or top 20 genes, whereas MYSM1 was identified only within the broader top 10% ranked cutoff.

We first examined whether the FDR-corrected coexpression network hubs represented TFs. Among the 251 final-ranked network genes, seven genes had TF annotation support based on the combined strict GO Molecular Function TF or HumanTFs definition: MAFB (MAF bZIP transcription factor B), MYB (MYB proto-oncogene, transcription factor), CCDC17 (coiled-coil domain containing 17), MYSM1 (Myb like, SWIRM and MPN domains 1), ZMAT1 (zinc finger matrin-type 1), ZNF491 (zinc finger protein 491), and ZNF789 (zinc finger protein 789). Four of these genes, MAFB, MYB, ZNF491, and ZNF789, were supported by strict GO TF annotation, whereas all seven were supported by the HumanTFs curated TF catalog. Five genes, MAFB, MYB, MYSM1, ZNF491, and ZNF789, were also classified as broad GO transcription regulators. In contrast, 244 of the 251 ranked network genes had no TF or broad transcription regulator annotation. Across the hub cutoff analysis, no TFs were detected among the top 0.5%, top 1%, top 5%, top 10 genes, or top 20 genes. Only MYSM1 appeared within the broader top 10%-ranked cutoff (Fig. 5). These results indicate that the leading degree-ranked hubs were predominantly network connectivity hubs rather than TF-annotated hubs.

We next evaluated TF annotation directly within the concordantly regulated shared DEG set. Seven of the 262 concordant shared DEGs were annotated as TFs using the combined strict GO or HumanTFs definition: MAFB, MYB, CCDC17, MYSM1, ZMAT1, ZNF491, and ZNF789 (Table 3). All seven TF annotated DEGs showed concordant directionality between CLL and MM. MAFB and MYB were upregulated in both diseases, whereas CCDC17, MYSM1, ZMAT1, ZNF491, and ZNF789 were downregulated in both diseases. MAFB showed log_2_ fold changes of 7.289 in CLL and 1.907 in MM, and MYB showed log_2_ fold changes of 5.485 in CLL and 2.607 in MM. The downregulated TF annotated DEGs also showed consistent negative directionality across both diseases, including CCDC17, MYSM1, ZMAT1, ZNF491, and ZNF789.

**Table 3:** Shared differentially expressed TFs identified in CLL and MM.

Gene	Strict GO TF	HumanTFs Curated TF	Broad GO Transcription Regulator	CLL Log_2_FC	MM Log_2_FC	Direction
MAFB	Yes	Yes	Yes	7.289	1.907	Upregulated in both
MYB	Yes	Yes	Yes	5.485	2.607	Upregulated in both
CCDC17	No	Yes	No	−1.163	−1.042	Downregulated in both
MYSM1	No	Yes	Yes	−1.263	−1.046	Downregulated in both
ZMAT1	No	Yes	No	−1.602	−2.039	Downregulated in both
ZNF491	Yes	Yes	Yes	−1.348	−1.136	Downregulated in both
ZNF789	Yes	Yes	Yes	−1.019	−1.474	Downregulated in both

Note: TF, transcription factor; GO, Gene Ontology; CLL, chronic lymphocytic leukemia; MM, multiple myeloma; log_2_FC, log_2_ fold change. Strict GO TF indicates support from GO Molecular Function transcription factor-related annotations. HumanTFs curated TF indicates membership in the HumanTFs/Lambert v1.01 curated human TF catalog. Broad GO transcription regulator indicates support from broader GO transcription regulator annotations. These annotations identify transcript-level TF candidates and do not establish TF protein activity, chromatin binding, or causal regulatory function.

To determine whether this TF overlap exceeded random expectation, we performed an expression-matched permutation analysis using the background-compatible subset of the concordantly regulated shared DEGs. Of the 262 concordant shared DEGs, 170 were compatible with the expressed background universe and were used in the permutation analysis. Using the combined strict GO TF or HumanTFs definition as the primary TF definition, seven TFs were observed among these 170 genes. The expression-matched null model expected an average of 13.13 TFs across 100,000 permutations using 20 expression bins. The observed TF count was therefore below expectation, with a fold over expected value of 0.533, a z score of minus 1.782, and an empirical enrichment *p* value of 0.9808. The empirical depletion *p*-value was 0.0424. Sensitivity analyses using 10- and 30-expression bins yielded similar expectations, supporting the same conclusion.

These TF analysis results (Fig. 5 and Table 3) identify a small, directionally concordant set of TF annotated DEGs within the shared CLL and MM transcriptomic signature, but they do not support global enrichment of TFs among the concordantly regulated shared DEGs. Therefore, MAFB, MYB, CCDC17, MYSM1, ZMAT1, ZNF491, and ZNF789 represent annotated, shared, differentially expressed TF candidates within the concordant transcriptomic and network context, whereas the overall results do not support broad TF enrichment or confirmed regulatory activity.

## 4 Discussion

This study provides a conservative cross-contextual transcriptomic analysis of CLL CD19-positive B cells and MM-associated bone marrow-derived mesenchymal stromal cells using publicly available bulk RNA sequencing datasets. Instead of directly comparing CLL and MM samples at baseline expression levels, differential expression was first defined separately within each disease context against its matched control group, followed by a summary-level comparison of shared DEGs, directionality, log_2_FC concordance, pathway enrichment, replicated coexpression structure, hub gene prioritization, and transcription factor annotation. The principal finding is that CLL CD19-positive B cells and MM-associated MSCs shared a focused concordantly regulated gene-level signature. Specifically, 323 significant DEGs overlapped between the two disease comparisons, and 262 of these showed the same direction of regulation: 52 genes were upregulated, and 210 were downregulated in both contexts. These concordantly regulated genes also showed strong alignment in log_2_FC structure between CLL and MM. In contrast, GO and KEGG enrichment analyses using an explicitly defined expressed-gene background did not identify statistically significant pathway-level enrichment after multiple testing correction. Therefore, the strongest evidence from this study supports a focused, cross-contextual, gene-level transcriptomic signature rather than a broad, canonical pathway-level mechanism or a validated shared regulatory program.

The detected DEG burden differed substantially between the two disease comparisons, with more DEGs identified in the CLL CD19-positive B-cell comparison than in the MM-associated MSC comparison. This difference should not be interpreted as evidence that CLL is globally more transcriptionally disrupted than MM, or that one disease context is biologically more active than the other. Instead, the difference likely reflects several overlapping factors, including sample size, cell source, disease biology, baseline transcriptional heterogeneity, and the nature of the analyzed cellular compartment. The CLL dataset was generated from CD19-positive B cells, which are closely aligned with the malignant B-cell compartment in CLL. In contrast, the MM dataset was generated from bone marrow-derived MSCs, which represent the tumor-associated stromal microenvironment rather than purified malignant plasma cells. MSCs are biologically heterogeneous, highly responsive to local inflammatory and tumor-derived signals, and may show greater within-group variability across donors. Therefore, the smaller DEG burden detected in the MM-associated MSC comparison may reflect stromal heterogeneity, microenvironmental diversity, and limited cohort size rather than a weaker role of the MM bone marrow niche. Accordingly, DEG counts in this study should be interpreted as dataset- and cell-context-specific detected transcriptional patterns, not as direct quantitative measures of disease severity, global transcriptional disruption, or biological importance.

A key interpretive step in this study was separating total shared DEG overlap from concordantly regulated shared DEGs. Although 323 significant DEGs were shared between the CLL CD19-positive B cell and MM-associated MSC comparisons, shared statistical significance alone does not necessarily indicate a common biological program. Genes that are significant in both datasets may still show opposite directions of regulation, reflecting disease-specific, cell-context-specific, microenvironment-related, or compensatory transcriptional responses. For this reason, the shared DEG pool was further classified by directionality. Among the 323 shared DEGs, 262 showed the same direction of regulation in both disease contexts, including 52 genes upregulated and 210 genes downregulated in both comparisons, whereas 61 genes showed opposite regulatory directions. This distinction was essential because the concordantly regulated subset provides a more biologically interpretable cross-contextual signature than total DEG overlap alone. The opposite-direction shared DEGs remain informative as discordant cross-context signals. But they were not used as evidence of shared transcriptional regulation. Therefore, the concordantly regulated DEG set served as the main basis for downstream log_2_FC concordance analysis, enrichment testing, replicated coexpression analysis, hub prioritization, and transcription factor annotation.

The log_2_FC concordance analysis provided additional support for the shared transcriptomic signature beyond DEG overlap and directionality alone. DEG overlap identifies genes that meet statistical thresholds in both disease comparisons, and directionality classification determines whether those genes change in the same regulatory direction across both comparisons. However, neither metric alone evaluates whether the magnitude of transcriptional change follows a similar structure across the two contexts. By comparing CLL and MM log_2_FC values across the 262 concordantly regulated shared DEGs, we found that the shared signature showed strong positive effect-size alignment. The high Pearson correlation indicated that genes with larger expression changes in one disease context tended to show larger changes in the other context, whereas the significant but more moderate Spearman correlation indicated that the exact rank order of individual genes was not fully preserved. This pattern is biologically reasonable given that the two datasets represent different cellular compartments, disease environments, and sample structures. Therefore, the concordantly regulated shared DEG set should be interpreted as a cross-contextual expression signature with aligned effect-size structure, but not as evidence that CLL CD19-positive B cells and MM-associated MSCs share identical transcriptional hierarchy or equivalent underlying mechanisms.

The pathway enrichment results further refined the interpretation of the concordantly regulated shared DEG signature. When GO and KEGG enrichment analyses were performed using an explicitly defined expressed-gene background universe, no terms remained statistically significant after Benjamini-Hochberg multiple testing correction. This result argues against interpreting the shared CLL CD19-positive B cell and MM-associated MSC signature as driven by a single dominant canonical pathway or a broadly enriched functional category. However, the absence of corrected pathway-level enrichment does not negate the gene-level concordance observed in the overlap, directionality, and log_2_FC analyses. Rather, it suggests that the shared signal identified here is more apparent at the level of specific concordantly regulated genes than at the level of broad GO or KEGG pathway overrepresentation. This distinction is important because enrichment results are sensitive to the size of the input gene list, gene annotation coverage, the background universe definition, and the structure of existing pathway databases [115]. Therefore, the negative enrichment result supports a more conservative interpretation. Indeed, the present study identifies a focused cross-contextual gene-level transcriptomic signature but does not provide strong evidence of a robust shared pathway-level mechanism between CLL CD19-positive B cells and MM-associated MSCs.

The coexpression analysis added an exploratory network-level layer to the concordantly regulated shared DEG signature while avoiding direct correlation analysis on a pooled cross-dataset expression matrix. Because the CLL and MM datasets were generated from different studies and distinct cellular compartments, coexpression networks were constructed independently within each dataset and then compared at the edge level. Among the 262 concordantly regulated shared DEGs, 254 genes were retained in both processed expression matrices and used for pairwise correlation testing. Edges were retained only if they met the predefined correlation and FDR thresholds independently in both the CLL and MM datasets and showed the same correlation sign in both contexts. This workflow identified 11,233 replicated same-sign edges involving 251 genes, suggesting that a large subset of the concordant DEG signature also showed reproducible coexpression structure across the two disease-associated contexts. However, because coexpression networks capture statistical associations among expression profiles rather than regulatory directionality or causal interactions [116], this network should be interpreted as exploratory evidence of coordinated expression behavior within the shared signature, not as proof of causal gene regulation, direct molecular interaction, or therapeutic dependency.

The highest-ranked hubs included PSMA3-AS1 (PSMA3 antisense RNA 1), SNORD58A (small nucleolar RNA, C/D box 58A), 100124516 (SNORD58C; small nucleolar RNA, C/D box 58C), MSS51 (MSS51 mitochondrial translational activator), 652966 (SNORD10; small nucleolar RNA, C/D box 10), 101928816 (LOC101928816; uncharacterized noncoding RNA locus), 100113381 (SNORD19B; small nucleolar RNA, C/D box 19B), 112268259 (LOC112268259; uncharacterized noncoding RNA locus), NKTR (natural killer cell triggering receptor), and SNORD38A (small nucleolar RNA, C/D box 38A). Several of these top-ranked nodes corresponded to noncoding RNAs, numerical identifiers, or less-characterized loci, indicating that the shared coexpression structure was not dominated by classical protein-coding oncogenic regulators. Because degree centrality reflects the number of replicated network edges connected to each gene [117], these hubs should be interpreted as highly connected expression features rather than causal drivers. The stability analyses strengthened this interpretation by showing that the leading hubs remained prominent across cutoff definitions, dense random network comparison, edge subsampling, and stricter edge-retention thresholds. These findings suggest that the top-ranked hubs were not explained by network density alone or by a small subset of unstable edges. Nevertheless, hub status in a coexpression network does not establish regulatory direction, protein-level function, disease dependency, or therapeutic relevance [118]. Therefore, the identified hubs should be considered exploratory network anchors within the concordantly regulated shared DEG signature and as candidates for future validation, rather than as confirmed mechanistic regulators of CLL or MM biology.

The transcription factor analysis further clarified the distinction between differential expression, network connectivity, and regulatory interpretation. Within the concordantly regulated shared DEG signature, seven genes were annotated as TF candidates using strict GO and/or HumanTFs-based definitions: MAFB, MYB, CCDC17, MYSM1, ZMAT1, ZNF491, and ZNF789 [119]. All seven showed concordant directionality between CLL CD19-positive B cells and MM-associated MSCs, with MAFB and MYB upregulated in both contexts and CCDC17, MYSM1, ZMAT1, ZNF491, and ZNF789 downregulated in both contexts. However, the highest-degree coexpression hubs were not dominated by TF-annotated genes, indicating that network centrality and transcription factor annotation represented distinct analytical features. Notably, MYSM1 was the only TF-annotated candidate detected within the broader top 10% degree-ranked network cutoff, although no TFs were present among the stricter top 10 or top 20 hub groups. This finding does not indicate that the coexpression network was broadly TF-driven, but it may still be biologically relevant because MYSM1 links transcriptional regulation, chromatin remodeling, hematopoiesis, B-cell development, and stromal cell biology [120,121,122]. Therefore, MYSM1 may represent a candidate point of intersection between the concordant shared DEG signature and the replicated coexpression structure. However, experimental validation is still required before any regulatory role can be inferred. Moreover, the expression-matched permutation analysis did not support global enrichment of TF annotations among the concordantly regulated shared DEGs. Therefore, these seven genes should be interpreted as specific shared differentially expressed TF candidates within the concordant transcriptomic signature, not as evidence that the overall shared signature is broadly TF-driven. Importantly, transcript-level TF annotation does not establish TF protein abundance, nuclear localization, chromatin binding, post-translational activation, or causal regulatory function [123]. Experimental validation would therefore be required before any of these TF candidates could be considered functional regulators of shared CLL- and MM-associated transcriptional contexts.

Among the shared differentially expressed TF candidates, MAFB and MYB are particularly notable because both were concordantly upregulated and are well established as relevant to hematopoietic lineage regulation, immune biology, and malignant blood cell programs. MAFB is linked to hematopoietic differentiation and has recognized relevance in plasma cell myeloma biology, whereas MYB is a major transcriptional regulator of hematopoiesis and B-cell development. Their concordant upregulation may therefore reflect activation of lineage-associated or disease-adaptive transcriptional states across the CLL B-cell and MM-associated stromal contexts. However, the interpretation of these genes must remain context-specific. In the CLL dataset, altered TF expressions may more directly reflect malignant or disease-associated B-cell transcriptional remodeling, whereas in the MM dataset, they may reflect transcriptional reprogramming of bone marrow-derived MSCs within the tumor-supportive niche rather than plasma cell-intrinsic regulation [124]. The concordantly downregulated TF candidates, including MYSM1, ZMAT1, ZNF491, ZNF789, and CCDC17, may indicate reduced expression of selected chromatin-associated, zinc-finger, or less-characterized regulatory genes within the shared signature. MYSM1 is particularly relevant given its established roles in hematopoiesis, B-cell development, chromatin regulation, and stromal cell biology [125]. In contrast, the functional relevance of ZMAT1, ZNF491, ZNF789, and CCDC17 to CLL or MM remains less established and should be considered hypothesis-generating.

**Figure 6:**
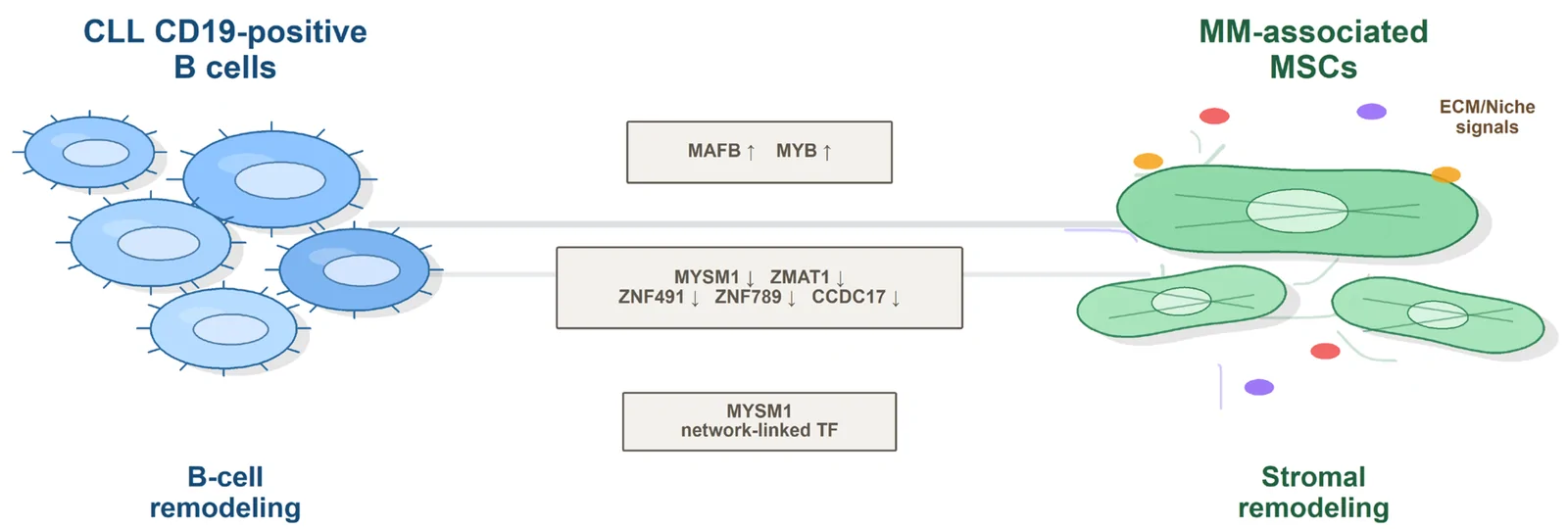
Hypothesis model of concordant shared transcriptional and TF-associated remodeling between CLL CD19-positive B cells and MM-associated mesenchymal stromal cells. The left side represents the CLL CD19-positive B-cell compartment and the associated B-cell remodeling context, whereas the right side represents the MM-associated MSC compartment, stromal remodeling, and ECM/Niche signals. The central boxes summarize annotated TF candidates within the concordantly regulated set of shared DEGs. MAFB and MYB are shown as upregulated TF candidates, while MYSM1, ZMAT1, ZNF491, ZNF789, and CCDC17 are shown as downregulated TF candidates. MYSM1 is separately highlighted as a network-linked TF candidate. The horizontal connecting lines indicate a conceptual cross-disease transcriptomic relationship and do not represent experimentally demonstrated physical cell–cell interaction. The model should be interpreted as a hypothesis-generating summary, in which the highlighted TF candidates may represent one potential regulatory layer contributing to the observed remodeling patterns, while additional regulatory, microenvironmental, epigenetic, and signaling mechanisms may also contribute to the observed B-cell and stromal remodeling patterns and require experimental validation.

In fact, these TF findings support a hypothesis-generating model [126] in which the shared CLL CD19-positive B-cell and MM-associated MSC transcriptomic signature may reflect a cross-contextual lineage–niche regulatory imbalance rather than a single shared oncogenic pathway. We summarize this interpretation schematically in Fig. 6, which integrates the concordant shared DEG pattern, annotated TF candidates, and network-linked MYSM1 finding into a conceptual CLL B-cell and MM-associated stromal remodeling framework. In this model, concordant upregulation of MAFB and MYB may represent an adaptive or disease-associated transcriptional arm linked to hematopoietic lineage regulation, B-cell activation biology, and plasma cell or bone marrow niche-associated remodeling. In parallel, concordant downregulation of MYSM1 and selected zinc-finger or less-characterized TF candidates may reflect reduced expression of regulatory factors involved in chromatin control, differentiation-associated transcriptional stability, or stromal homeostatic programs. MYSM1 is especially relevant to this model because it was the only TF-annotated gene detected within the broader top 10% degree-ranked network cutoff and has established roles in hematopoiesis, B-cell development, chromatin regulation, and MSC biology. Therefore, we hypothesize that the shared TF pattern observed here may mark a disease-adaptive transcriptional state at the interface of malignant B-cell biology in CLL and tumor-supportive stromal remodeling in MM. This hypothesis does not imply that CLL cells and MM-associated MSCs share identical mechanisms, nor does it suggest that the observed remodeling patterns are driven exclusively by the highlighted TF candidates. Additional regulatory, microenvironmental, epigenetic, extracellular matrix-associated, and signaling mechanisms may also contribute to these remodeling patterns and require experimental validation. Accordingly, this model does not establish TF activity or causality while it provides a testable framework for future studies using matched malignant plasma cell and stromal datasets, single-cell or spatial transcriptomics, chromatin accessibility profiling, TF perturbation assays, and protein-level validation to determine whether MAFB, MYB, MYSM1, or the downregulated zinc-finger candidates contribute functionally to shared hematologic malignancy-associated transcriptional remodeling.

## 5 Study Limitations

Our findings should be interpreted in light of several limitations.

We used two publicly available bulk RNA sequencing datasets from distinct cellular compartments. The CLL dataset represents CD19-positive B cells, whereas the MM dataset represents bone marrow-derived MSCs rather than malignant plasma cells. Therefore, the shared DEG signature should not be interpreted as direct transcriptional equivalence between CLL cells and MM plasma cells, nor as definitive evidence of MM tumor-intrinsic transcriptional regulation. Instead, the results identify candidate cross-contextual disease-associated signals spanning malignant B-cell biology in CLL and tumor-supportive stromal remodeling in MM. Validation using matched malignant plasma cell datasets, stromal datasets, and experimentally harmonized cellular models will be required to distinguish tumor-intrinsic mechanisms from microenvironment-associated transcriptional programs.

Our study was based on a single public RNA sequencing dataset per disease and modest sample sizes in both cohorts. Although the selected datasets provided human primary samples, disease and control groups, count-level RNA sequencing data, and biologically relevant cell sources, the analysis was not prospectively powered to detect all disease-associated transcriptional changes. Weaker, more heterogeneous, or context-specific signals may therefore have been missed. Conservative DEG thresholds, directionality classification, log_2_FC concordance analysis, and cross-context filtering helped reduce overinterpretation, but these measures do not replace validation in larger independent cohorts.

Batch effects and inter-study heterogeneity remain important limitations. The CLL and MM datasets were generated in independent studies and may differ in cohort composition, sample handling, library preparation, sequencing platform, laboratory processing, and other unreported technical factors. To reduce this risk, raw counts were not pooled for a direct cross-dataset differential expression model. Instead, disease-versus-control contrasts were estimated independently within each dataset, and cross-disease interpretation was restricted to summary-level comparisons. Nevertheless, because disease context, cell source, study origin, and technical factors cannot be fully separated in this retrospective public dataset design, the identified shared signals should be considered hypothesis-generating.

Surrogate variable adjustment was not incorporated into the primary raw count-based DESeq2 workflow. Instead, exploratory SVA-based sensitivity analyses were performed using processed expression matrices. These analyses suggested that the overall log_2_FC structure and directionality of disease-associated signals were largely preserved, particularly in the CLL dataset, whereas threshold-based DEG classification in the smaller MM-associated MSC dataset was more sensitive to adjustment for surrogate variables. Therefore, residual latent technical or cohort-related variation cannot be entirely ruled out, especially in the MM analysis.

The coexpression, hub gene, and transcription factor analyses should be interpreted as exploratory prioritization layers rather than mechanistic validation. Although coexpression networks were constructed independently within each dataset and the final network retained only replicated same-sign edges observed in both contexts, coexpression still represents statistical association rather than regulatory direction, physical interaction, or causality. Similarly, degree-ranked hub genes identify highly connected network nodes but do not establish disease dependency, protein-level function, or therapeutic relevance. Therefore, the hub findings require validation using independent expression datasets, perturbation experiments, and functional assays.

Finally, the TF findings remain candidate observations at the transcript level. TF annotation identified biologically interpretable candidates, including MAFB, MYB, MYSM1, ZMAT1, ZNF491, ZNF789, and CCDC17, but mRNA abundance and annotation status do not establish TF protein abundance, nuclear localization, chromatin binding, post-translational activation, or causal regulatory function. In addition, the proposed lineage–niche regulatory imbalance model is a hypothesis-generating interpretation based on the observed concordant TF pattern and requires experimental validation. Future studies using independent cohorts, matched malignant plasma cell and stromal datasets, single-cell or spatial transcriptomics, chromatin accessibility profiling, protein-level TF assessment, and perturbation-based models will be needed to determine whether these candidate TFs functionally contribute to shared disease-associated transcriptional remodeling in CLL and MM-related contexts.

## 6 Conclusion

This study identified a focused cross-contextual transcriptomic signature shared between CLL CD19-positive B cells and MM-associated bone marrow-derived MSCs. By analyzing each disease context against its matched control group and then evaluating shared DEG directionality, log_2_FC concordance, coexpression structure, hub connectivity, and TF annotation, the study provides a conservative framework for comparing disease-associated transcriptional patterns across distinct hematologic compartments. The concordantly regulated subset of DEGs showed aligned expression changes between CLL CD19-positive B-cells and MM-associated MSCs. Network analysis further identified exploratory coexpression anchors within this shared signature, while TF annotation highlighted biologically interpretable candidates, including MAFB, MYB, MYSM1, ZMAT1, ZNF491, ZNF789, and CCDC17. Together, these findings suggest partial convergence of transcriptional regulatory programs between CLL malignant B-cell states and MM-associated stromal remodeling, despite derivation from distinct hematopoietic and mesenchymal lineages with independent fate trajectories. Thus, the observed TF pattern may reflect coordinated dysregulation of lineage-specific and microenvironment-associated regulatory programs underlying malignant B-cell remodeling in CLL and tumor-supportive stromal remodeling in MM. Future studies should pursue: (i) validation of the shared transcriptional signature in independent CLL and MM patient cohorts to assess reproducibility and generalizability; (ii) integration of matched plasma cell and bone marrow stromal multi-omics datasets to refine compartment-specific regulatory programs; (iii) single-cell RNA-seq and spatial transcriptomic profiling to resolve cell-type–specific and spatially organized lineage–niche interactions; (iv) functional perturbation of prioritized TFs using CRISPR-based knockdown, activation, or knockout approaches to establish causal regulatory roles; (v) epigenomic profiling to characterize chromatin accessibility landscapes underlying the shared transcriptional programs; (vi) ligand–receptor interaction analysis and intercellular communication modeling to investigate stromal–tumor signaling axes; (vii) longitudinal sampling of disease progression cohorts to determine temporal stability and evolution of the shared regulatory signature; and (viii) multi-layer network reconstruction integrating transcriptomic, epigenomic, and regulatory inference approaches to identify upstream master regulators driving the observed cross-disease concordance.

## Data Availability

The main data supporting the findings of this study are available within the paper and its Supplementary Information. The datasets used and analyzed during the current study are available from the corresponding author on reasonable request.

## Supplementary Materials

The supplementary material is available online at https://www.techscience.com/doi/10.32604/or.2026.082424/s1.

## Abbreviations

**Abbreviation/Symbol**	**Meaning**
<	Less than
>	Greater than
≥	Greater than or equal to
|r|	Absolute value of the Pearson correlation coefficient
ρ	Spearman’s rank correlation coefficient
×	Multiplication sign
−	Minus sign
β2	Beta-2
ATM	ATM serine/threonine kinase
BCL-2	B-cell lymphoma 2
BCR	B-cell receptor
BRAF	B-Raf proto-oncogene, serine/threonine kinase
CCDC17	Coiled-coil domain containing 17
CD5	Cluster of differentiation 5
CD19	Cluster of differentiation 19
CD20	Cluster of differentiation 20
CD23	Cluster of differentiation 23
CLL	Chronic lymphocytic leukemia
clusterProfiler	R/Bioconductor package for functional enrichment analysis
CPM	Counts per million
CSV	Comma-separated values
DEG/DEGs	Differentially expressed gene(s)
del	Chromosomal deletion
DESeq2	Differential expression analysis package for sequence count data
DNA	Deoxyribonucleic acid
ECM	Extracellular matrix
Entrez ID	Entrez Gene identifier
FDR	False discovery rate
GEO	Gene Expression Omnibus
ggplot2	R package for data visualization
GO	Gene Ontology
GO.db	Gene Ontology annotation database package
GOALL	Gene Ontology annotations including direct and ancestor terms
GSE	Gene Expression Omnibus Series accession
H/ACA	H/ACA box small nucleolar RNA class
HumanTFs	Curated catalog of human transcription factors
ID	Identifier
iDEP	Integrated Differential Expression and Pathway Analysis
IEA	Inferred from Electronic Annotation
igraph	R package for network and graph analysis
INE1	Inactivation escape 1
ISS	International Staging System
KEGG	Kyoto Encyclopedia of Genes and Genomes
KRAS	KRAS proto-oncogene, GTPase
limma	Linear Models for Microarray Data R/Bioconductor package
log_2_FC	log_2_ fold change
MAFB	MAF bZIP transcription factor B
MASP2	MBL-associated serine protease 2
MM	Multiple myeloma
MSC/MSCs	Mesenchymal stromal cell(s)
MSS51	Mitochondrial translational activator
MYB	MYB proto-oncogene, transcription factor
MYSM1	MYB-like, SWIRM and MPN domains 1
N	Number of libraries or filtering threshold parameter, depending on context
n	Number of samples
NCBI	National Center for Biotechnology Information
NF-κB	Nuclear factor kappa B
NIH	National Institutes of Health
NKTR	Natural killer cell triggering receptor
NLM	National Library of Medicine
NRAS	NRAS proto-oncogene, GTPase
NSU	North South University
num.sv	R function used to estimate the number of surrogate variables
org.Hs.eg.db	Genome-wide human annotation package
P/p	*p*-value
PC1	First principal component
PC2	Second principal component
PCA	Principal component analysis
prcomp	R function used for principal component analysis
PSMA3-AS1	PSMA3 antisense RNA 1
r	Pearson correlation coefficient
R	R programming language
R-ISS	Revised International Staging System
RNA	Ribonucleic acid
RNA-seq	RNA sequencing
RStudio	Integrated development environment for R
SF3B1	Splicing factor 3b subunit 1
SNORA17B	Small nucleolar RNA, H/ACA box 17B
SNORD38A	Small nucleolar RNA, C/D box 38A
SNORD58A	Small nucleolar RNA, C/D box 58A
SVA	Surrogate variable analysis
TF/TFs	Transcription factor(s)
TP53	Tumor protein p53
UAB	Universitat Autònoma de Barcelona
UOC	Universitat Oberta de Catalunya
UPV	Universitat Politècnica de València
VST	Variance stabilizing transformation
ZMAT1	Zinc finger matrin-type 1
ZNF491	Zinc finger protein 491
ZNF789	Zinc finger protein 789
