# Supplementary material for: Concordant Shared Transcriptomic Signatures and Candidate Regulatory Features in Chronic Lymphocytic Leukemia and Multiple Myeloma
Source: Oncol Res. 2026 Aug 13;34(9):21. doi: 10.32604/or.2026.082424 (PMC13494498; doi:10.32604/or.2026.082424)
Supplement: Supplementary file 1 [file OncolRes-34-82424-s001.zip › Supplementary Fig. S1.docx]

**Supplementary Figure S1:** Mean-variance plots for the CLL and MM RNA-seq datasets.

(A) CLL dataset; (B) MM dataset. Each point represents a gene, with the x-axis showing mean expression and the y-axis showing standard deviation across samples. The blue trend line summarizes the relationship between mean expression and variability, and the color scale indicates local point density/count. These plots were used to evaluate the mean-variance relationship before downstream exploratory analyses. CLL, chronic lymphocytic leukemia; MM, multiple myeloma; RNA-seq, RNA sequencing.
