# Supplementary material for: Concordant Shared Transcriptomic Signatures and Candidate Regulatory Features in Chronic Lymphocytic Leukemia and Multiple Myeloma
Source: Oncol Res. 2026 Aug 13;34(9):21. doi: 10.32604/or.2026.082424 (PMC13494498; doi:10.32604/or.2026.082424)
Supplement: Supplementary file 1 [file OncolRes-34-82424-s001.zip › Supplementary Fig. S2.docx]

**Supplementary Figure S2:** Scree plots for the CLL and MM RNA-seq datasets.

(A) CLL dataset; (B) MM dataset. Bars indicate the percentage of variation explained by each principal component, and the red line indicates cumulative explained variation. Dashed vertical lines mark the component-selection points identified using Horn’s parallel analysis and the elbow criterion. These plots were used to assess the variance structure of the transformed expression matrices prior to downstream exploratory analyses. CLL, chronic lymphocytic leukemia; MM, multiple myeloma; RNA-seq, RNA sequencing.
