# Supplementary material for: Concordant Shared Transcriptomic Signatures and Candidate Regulatory Features in Chronic Lymphocytic Leukemia and Multiple Myeloma
Source: Oncol Res. 2026 Aug 13;34(9):21. doi: 10.32604/or.2026.082424 (PMC13494498; doi:10.32604/or.2026.082424)
Supplement: Supplementary file 1 [file OncolRes-34-82424-s001.zip › Supplementary Fig. S3.docx]

**Supplementary Figure S3:** PCA plots for the CLL and MM RNA-seq datasets.

(A) Principal component analysis plot for the CLL dataset. (B) Principal component analysis plot for the MM dataset. PCA was performed on variance-stabilized transformed expression data to evaluate sample-level structure and exploratory separation between disease and control samples. Each point represents one sample, and the axes indicate the first two principal components. CLL, chronic lymphocytic leukemia; MM, multiple myeloma; PCA, principal component analysis; RNA-seq, RNA sequencing.
