# Supplementary material for: Concordant Shared Transcriptomic Signatures and Candidate Regulatory Features in Chronic Lymphocytic Leukemia and Multiple Myeloma
Source: Oncol Res. 2026 Aug 13;34(9):21. doi: 10.32604/or.2026.082424 (PMC13494498; doi:10.32604/or.2026.082424)
Supplement: Supplementary file 1 [file OncolRes-34-82424-s001.zip › Supplementary Fig. S4.docx]

**Supplementary Figure S4:** Pathway enrichment mapping and corrected significance summary.

(A) Explicitly expressed background universe used for GO and KEGG enrichment analyses showing the intersection of post-filtered CLL and MM expression matrices and the mapped Entrez Gene ID background used for overrepresentation testing. (B) Concordant DEG mapping and background retention for genes upregulated in both diseases, downregulated in both diseases, and the combined concordant DEG pool. The panel summarizes the number of input gene symbols, mapped Entrez IDs, and Entrez IDs retained within the explicitly expressed background universe. (C) Corrected enrichment significance summary for the combined concordant DEG pool evaluated against the explicitly expressed background. GO and KEGG analyses returned enrichment terms. But no terms remained statistically significant after Benjamini-Hochberg correction (BH < 0.05 = 0). These results indicate that the shared CLL and MM transcriptomic signal was not dominated by robust pathway-level overrepresentation under the dataset-specific expressed background definition. BH, Benjamini-Hochberg; CLL, chronic lymphocytic leukemia; DEG, differentially expressed gene; GO, Gene Ontology; KEGG, Kyoto Encyclopedia of Genes and Genomes; MM, multiple myeloma.
