# Supplementary material for: Concordant Shared Transcriptomic Signatures and Candidate Regulatory Features in Chronic Lymphocytic Leukemia and Multiple Myeloma
Source: Oncol Res. 2026 Aug 13;34(9):21. doi: 10.32604/or.2026.082424 (PMC13494498; doi:10.32604/or.2026.082424)
Supplement: Supplementary file 1 [file OncolRes-34-82424-s001.zip › Supplementary Table S1.docx]

**Supplementary Table S1:** Summary of the clinical and selection criteria for the CLL samples

| **Field** | **Information** |
| --- | --- |
| Dataset | GSE70830 |
| Cohort | 10 CLL samples and 5 normal control samples |
| Sample source | Peripheral blood CD19-positive B cells |
| CLL patient status | Untreated CLL patients |
| Ethics and consent | Samples were obtained after institutional human subject approval and written informed consent, as reported in the associated publication |
| Available clinical information | Age, sex, Rai stage, white blood cell count, CD38 positivity, ZAP70 status, and IGVH mutation status |
| Control samples | Normal peripheral blood CD19-positive B cell controls |
| Inclusion basis for the present study | Publicly available RNA seq data, CLL disease status, untreated patient status, peripheral blood CD19-positive B cell origin, and availability of normal CD19-positive B cell controls |
| Exclusion criteria | Formal exclusion criteria were not specified in the public GEO record or associated publication |
| Source | Public GEO record and associated publication |

Note: CLL, chronic lymphocytic leukemia; GEO, Gene Expression Omnibus; IGVH, immunoglobulin heavy-chain variable region; RNA-seq, RNA sequencing; ZAP70, zeta-chain-associated protein kinase 70. Clinical and sample-selection information was summarized from the public GEO record and the associated publication.
